# Glycerol-derived organic carbonates: environmentally friendly plasticizers for PLA†

**DOI:** 10.1039/d3ra08922c

**Published:** 2024-02-05

**Authors:** Hyeon Jeong Seo, Yeong Hyun Seo, Sang Uk Park, Hyun Ju Lee, Mi Ryu Lee, Jun Hyeong Park, Woo Yeon Cho, Pyung Cheon Lee, Bun Yeoul Lee

**Affiliations:** a Department of Molecular Science and Technology, Ajou University Suwon 16499 South Korea bunyeoul@ajou.ac.kr pclee@ajou.ac.kr +82-31-219-2394 +82-31-219-1844

## Abstract

Polylactic acid (PLA) stands as a promising material, sourced from renewables and exhibiting biodegradability—albeit under stringent industrial composting settings. A primary challenge impeding PLA's broad applications is its inherent brittleness, as it fractures with minimal elongation despite its commendable tensile strength. A well-established remedy involves blending PLA with plasticizers. In this study, a range of organic carbonates—namely, 4-ethoxycarbonyloximethyl-[1,3]dioxolan-2-one (1), 4-methoxycarbonyloximethyl-[1,3]dioxolan-2-one (2), glycerol carbonate (3), and glycerol 1-acetate 2,3-carbonate (4)—were synthesized on a preparative scale (∼100 g), using renewable glycerol and CO_2_-derived diethyl carbonate (DEC) or dimethyl carbonate (DMC). Significantly, 1–4 exhibited biodegradability under ambient conditions within a week, ascertained through soil exposure at 25 °C—outpacing the degradation of comparative cellulose. Further investigations revealed 1's efficacy as a PLA plasticizer. Compatibility with PLA, up to 30 phr (parts per hundred resin), was verified using an array of techniques, including DSC, DMA, SEM, and rotational rheometry. The resulting blends showcased enhanced ductility, evident from tensile property measurements. Notably, the novel plasticizer 1 displayed an advantage over conventional acetyltributylcitrate (ATBC) in terms of morphological stability. Slow crystallization, observed in PLA/ATBC blends over time at room temperature, was absent in PLA/1 blends, preserving amorphous domain dimensions and mitigating plasticizer migration—confirmed through DMA assessments of aged and unaged specimens. Nevertheless, biodegradation assessments of the blends revealed that the biodegradable organic carbonate plasticizers did not augment PLA's biodegradation. The PLA in the blends remained mostly unchanged under ambient soil conditions of 25 °C over a 6 month period. This work underscores the potential of organic carbonates as both eco-friendly plasticizers for PLA and as biodegradable compounds, contributing to the development of environmentally conscious polymer systems.

## Abstract 2

Synopsis: A organic carbonate derived from glycerol, ethanol, and CO_2_ demonstrates efficient plasticizing properties for PLA, offering a notable advantage in terms of morphological stability over the conventional plasticizer, acetyltributylcitrate.

## Graphical abstract

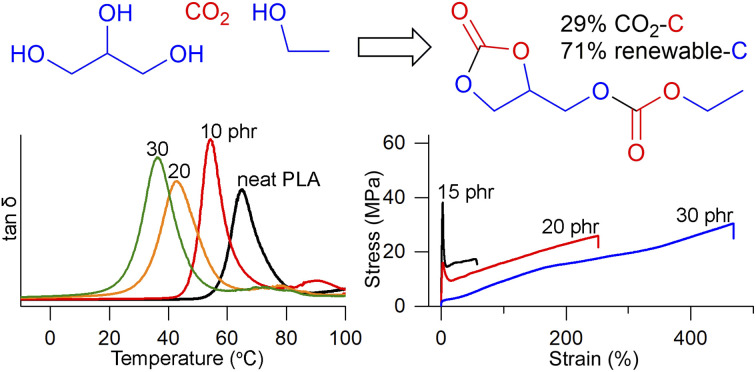

## Introduction

**Scheme 1:**
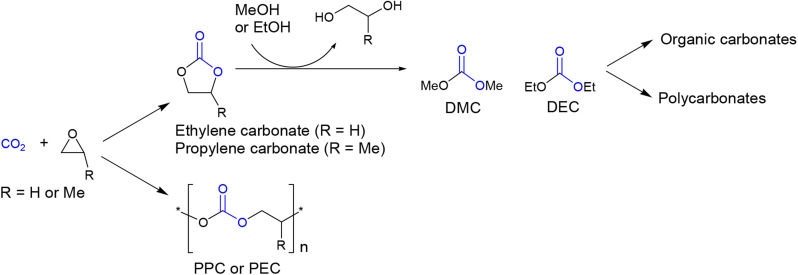
Exploiting CO_2_ for the synthesis of organic carbonates and polycarbonates.

Organic carbonates are valuable compounds with diverse applications,^1^ including benign solvents,^2,3^ lithium-ion battery electrolytes,^4^ fuel additives,^5,6^ and polymer synthesis.^7,8^ Traditionally, organic carbonates were prepared using highly toxic phosgene. However, currently, a more attractive method involves their production from CO_2_.^9–11^ One established process for synthesizing organic carbonates involves the coupling reaction of CO_2_ with ethylene oxide or propylene oxide, yielding ethylene carbonate or propylene carbonate (Scheme 1).^12^ These compounds can then be further converted to DMC or DEC while simultaneously producing anhydrous-grade ethylene glycol or propylene glycol.^13–15^ DMC or DEC can also serve as starting materials for the production of other organic carbonates through transcarbonation reactions with various alcohols.^16,17^ Additionally, they can be used as raw materials for the production of conventional bisphenol-A-based aromatic polycarbonates as well as biodegradable aliphatic polycarbonates.^18–21^ The production of organic carbonates and aliphatic polycarbonates holds great promise in the field of CO_2_ capture and utilization (CCU) technology.^22–28^

On the other hands, there has been recently a significant increase in interest regarding biodegradable polymers, particularly those manufactured using renewable resources.^29,30^ PLA serves as a typical example, but its annual production remains insignificant, amounting to only several hundred kilotons, when compared to the total polymer market size (∼300 megatons per year). One obstacle to the market expansion of PLA could be its non-biodegradability under ambient conditions, such as soil, fresh water, marine environments, and home composting, although it does biodegrade in industrial composting sites operating under harsh artificial conditions.^31^ Another challenge is its inherent brittleness.^32^ While PLA exhibits high tensile strength (approximately 60 MPa), its strain at break is low (around 5%). To address this issue, blending PLA with low molar mass organic compounds, commonly referred to as plasticizers, has been explored. Various ester compounds derived from citric acid,^33,34^ levulinic acid,^35–37^ tartaric acid,^38^ adipic acid,^39,40^ phthalic acid,^41^ glycerol,^42–44^ glucose,^45^ isosorbide,^46^ and lactones (*e.g.*, oligolactide)^47,48^ as well as some ether compounds such as poly(ethylene glycol)^49^ and epoxidized fatty oil have been attempted as plasticizers for PLA to impart ductile properties and overcome brittleness.^50–53^ When selecting a plasticizer for PLA, compatibility is a crucial criterion, along with other factors such as non-volatility (boiling temperature > 300 °C), non-toxicity, non-fuming, and odorlessness. Ideally, the plasticizer itself should be biodegradable and manufactured using renewable resources, further enhancing its desirability. In this study, we demonstrate the efficacy of organic carbonates derived from CO_2_ and renewable glycerol as PLA plasticizers. Our findings reveal that these organic carbonates remain intact during the blending process at high temperatures, while exhibiting rapid biodegradation under ambient soil conditions.

## Results and discussion

### Preparation of glycerol based organic carbonates

**Scheme 2:**
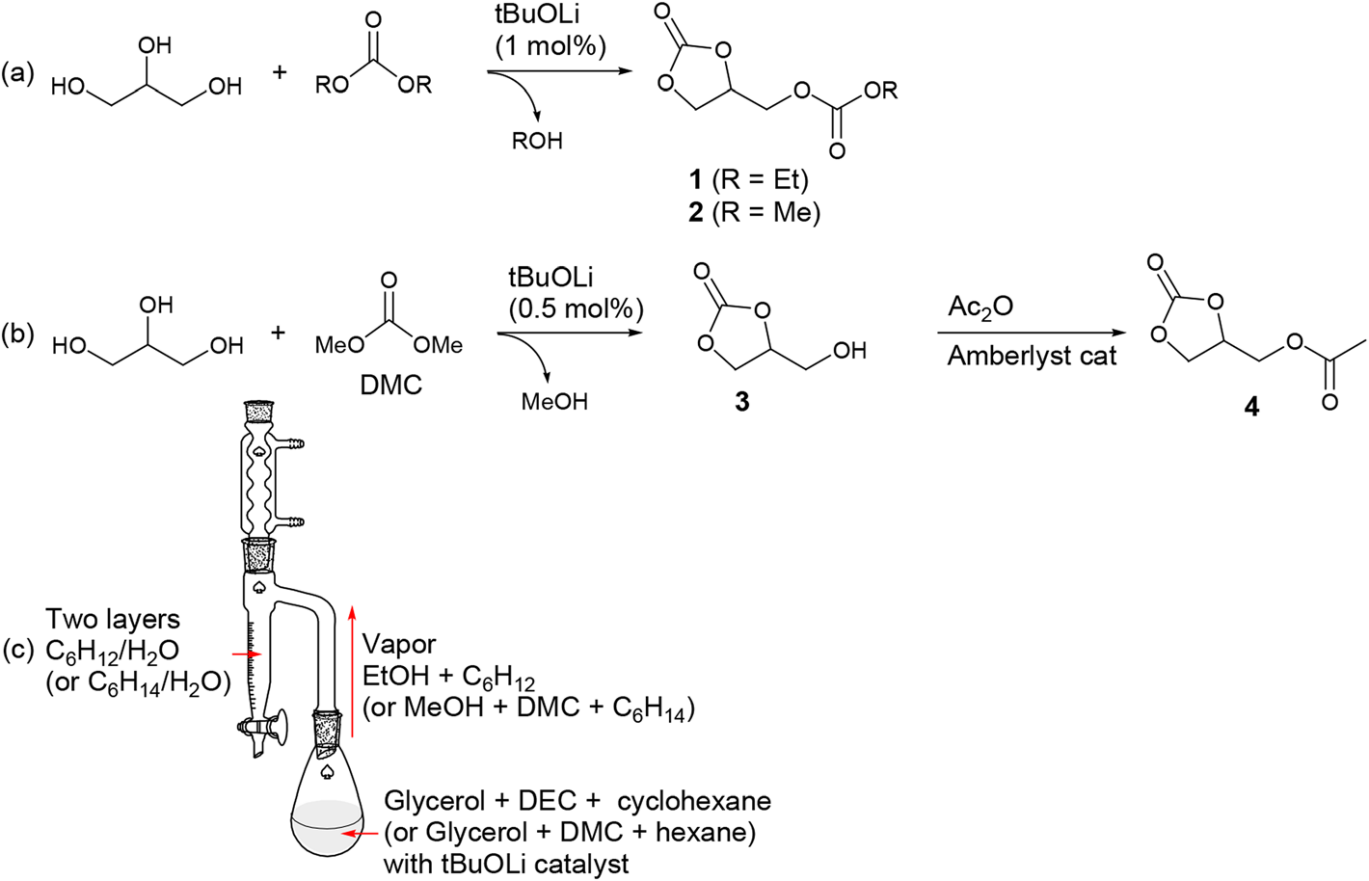
(a) and (b) Synthetic routes and (c) experimental setup for glycerol-based organic carbonates.

4-Ethoxycarbonyloximethyl-[1,3]dioxolan-2-one (1) is an appealing compound that can potentially be synthesized from CO_2_, glycerol, and ethanol. Glycerol and ethanol are representative renewable carbon sources, with the former being a byproduct of biodiesel production and the latter obtained through fermentation of sugars.^54^ However, its synthesis has been rarely reported. A century ago, it was reported to be prepared from glycerol using the toxic ethyl chloroformate (EtOC(O)Cl) and a stoichiometric amount of Na metal.^55^ The yield was very low (15%), and its boiling temperature was reported to be 304–306 °C. In our study, we successfully synthesized it on a 90 g-scale with a high yield (81%) using DEC (Scheme 2a). However, it's worth noting that the reaction and process conditions, as discussed below, still posed substantial challenges when it comes to cost-effective large-scale production. The product is formed through transcarbonation reactions between glycerol and DEC, catalyzed by lithium alkoxide (1.0 mol% per glycerol). It should be noted that the transcarbonation reaction is a reversible process, and to shift the equilibrium towards the product, the generated byproduct EtOH needs to be continuously removed from the reaction mixture. To facilitate this, we designed a reactor employing the Dean–Stark apparatus, which effectively removes the generated EtOH during the reaction (Scheme 2c).^16^ EtOH is distilled from the reaction mixture along with the carrier solvent cyclohexane, and both compounds are condensed in the Dean–Stark trap filled with two phases of cyclohexane and water. EtOH diffuses into the lower water phase while cyclohexane returns back into the reactor. Despite our efforts to minimize the undesired formation of diglycerol tricarbonate by using an excess of DEC (7 eq per glycerol), a small amount of diglycerol tricarbonate formation might have been unavoidable. However, we were able to eliminate this side product through vacuum distillation, resulting in the desired product 1 containing 1.5 mol% glycerol carbonate (Fig. S1†) with a yield of 81%, which was not quantitative but still satisfactory.

Using the developed reactor, we successfully prepared high-purity 4-methoxycarbonyloximethyl-[1,3]dioxolan-2-one (2) on a 60 g-scale (Fig. S2†). During the reaction, a side product, diglycerol tricarbonate, could also be generated, which reduced the isolated yield to 64%. Product 2 precipitated from the reaction mixture and was isolated by filtration, leaving the side product in the solution phase. In this case, the reactant DMC (boiling point, 90 °C) was distilled from the reaction mixture, forming an azeotrope with the byproduct MeOH. However, selective separation of MeOH was achieved in the Dean–Stark trap, while DMC, along with the carrier solvent hexane, returned back into the reactor. It is important to use anhydrous grades of reactants (glycerol, DEC, and DMC) for successful synthesis. Otherwise, glycerol may not be completely converted to the desired products 1 and 2, with some portion persistently remaining as the intermediate glycerol carbonate (3). Water can act as a source of catalyst poison, transforming the active lithium alkoxide (ROLi) catalyst into inactive lithium alkyl carbonate species (ROCO_2_Li). A preparative-scale synthesis of 2 using glycerol, DMC (10 eq), and K_2_CO_3_ (10 mol%) as the catalyst has also been reported. In that study, byproduct MeOH was continuously removed through azeotropic distillation with DMC, which was fed in excess, using a Vigreux column.^56^ We encountered a significant challenge when attempting to reproduce the synthesis of 1 and 2. In some instances, glycerol was not completely converted into the desired products 1 and 2, a portion persistently remaining as the intermediate glycerol carbonate (3) even after extended reaction times. Eventually, we found that using anhydrous-grade reactants (glycerol, DEC, and DMC) was crucial for achieving reproducibility. We suspect that water may serve as a source of catalyst poisoning, potentially transforming the active lithium alkoxide (ROLi) catalyst into inactive lithium alkyl carbonate species (ROCO_2_Li).

Extensive efforts were made to synthesize glycerol carbonate (3) due to its potential applications in polyurethane synthesis and as a fuel additive.^5,57^ Various heterogeneous and homogeneous catalysts have been tested for the transcarbonation reaction of glycerol and DMC to produce 3.^58^ For example, a reaction condition was established to achieve a 98% yield of 3 with 99% conversion. This was accomplished by reacting glycerol with 4 eq DMC for 2.5 h at refluxing temperature, utilizing a 10 mol% triethylamine catalyst.^56^ In this study, a 150 g-scale synthesis of 3 was carried out by reacting glycerol with 3 eq DMC using a much smaller amount (0.5 mol%) of a simple lithium alkoxide catalyst, ultimately yielding 99% isolated yield (Scheme 2b). The catalyst was easily removed by filtration, followed by a reaction with the ion exchange resin Amberlyst^®^. In this particular case, it was unnecessary to remove the generated MeOH during the reaction, as the formation of the 5-membered cyclic carbonate from DMC is an irreversible process and thermodynamically favorable. Some side product 2 (6.5 mol%) and residual reactant glycerol (5.0 mol%) persisted in the reaction mixture along with the desired product. However, during the distillation process, which was performed to remove the byproduct MeOH and excess DMC particularly in the presence of the lithium alkoxide catalyst, most of these impurities were converted to the desired product, resulting in the presence of 2.3 mol% of glycerol and 1.3 mol% of 2 (Fig. S3†). The synthesized glycerol carbonate was then transformed into glycerol 1-acetate 2,3-carbonate (4) by treating it with 1.2 eq acetic anhydride in the presence of an Amberlyst^®^ catalyst under neat conditions (Fig. S4†).

### Blending the organic carbonates with PLA

**Fig. 1:**
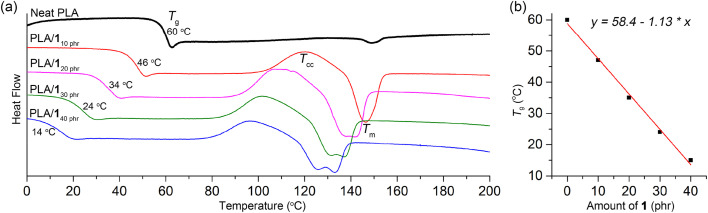
(a) Second-heating DSC curves of PLA/1 blends compared with neat PLA. (b) Linear correlation between the content of 1 and *T*_g_ of the blends.

Previous report indicate that propylene carbonate is miscible with PLA and can function as a plasticizer for PLA.^59^ However, its boiling temperature of 242 °C is not sufficiently high for practical use as a plasticizer. During storage under ambient conditions, we observed a slow escape of propylene carbonate from the polymer matrix. Specifically, initially transparent specimens, fabricated using a blend containing 20 phr of propylene carbonate, became hazy after being stored at room temperature for one month. Furthermore, their ductile properties deteriorated significantly, leading to a notable decrease in elongation at break, which went from 310 ± 190% to 35 ± 3%. The miscibility of polymers with organic compounds is highly dependent on their structure. When attempting to blend PLA with other organic carbonates such as ethylene carbonate and 3, phase separation was observed. To our delight, we found that 1 exhibited good miscibility with PLA, as demonstrated by a significant decrease in the glass transition temperature (*T*_g_) upon blending (Table 1). The *T*_g_ values gradually decreased from neat PLA value of 60 °C to 46, 34, 24, and 14 °C as the amount of 1 increased (10, 20, 30, and 40 phr, respectively). Notably, this decrease displayed a linear trend empirically (Fig. 1). To elaborate further, the *T*_g_ values of the blends conformed well to the Fox equation: 1/*T*_g-blend_ = *w*_PLA_/*T*_g-PLA_ + *w*_1_/*T*_g-__1_, where *w*_PLA_ and *w*_1_ represent the weight fractions of PLA and 1, respectively. The calculated *T*_g_ values, estimated using this equation with the measured *T*_g_ values of neat PLA and 1 (333 and 220 K, respectively; Fig. S5†), closely matched the values determined from the DSC curves (318, 307, 298, and 290 K, compared to the measured values of 319, 307, 297, and 287 K, respectively). Under the same amount of plasticizer, the *T*_g_ values of the PLA/1 blends were similar to those of the PLA/ATBC blend (34 *vs.* 30 °C at 20 phr; Fig. S5†) as well as the blend of oligolactide EtO[C(O)CH(Me)O]_4.5_C(O)CH_3_ (46 *vs.* 45 °C at 10 phr).^33,47^ This similarity is noteworthy, considering that the *T*_g_ value of ATBC is significantly lower than that of 1 (−84 °C *versus* −53 °C). The *T*_g_ curve was fairly distinct for the PLA blends containing 10–30 phr of 1, while the curve was rather blurred for the blend containing 40 phr of 1 (denoted as PLA/1_40 phr_). PLA/ATBC blends displayed a relatively more blurred transition curve than the PLA/1 blends under the same amount of plasticizer. 2 and 4 also exhibited miscibility with PLA, showing a similar linear dependency of the *T*_g_ values on the amounts of 2 and 4 (Fig. S7 and S8†).

**Table 1:** Thermal and mechanical properties of PLA/1, PLA/2, and PLA/4 blends compared to PLA/ATBC blends

Plasticizer (phr)	*T*_g_ (°C)	*T*_cc_ (°C); Δ*H* (J g^−1^)	*T*_m_ (°C); Δ*H* (J g^−1^)	tan *δ* peak *T*_g_ (°C)	E′′ peak *T*_g_ (°C)	E′ at 25 °C (MPa)	E′′ at 25 °C (MPa)	Tensile strength (MPa)	Strain at break (%)
PLA	60	—	148–156; 0.2	65	59	1600	17	60 ± 2	3 ± 0.1
1 (10)	46	103–125; 12	140–153; 12	54	48	1500	27	50 ± 4	3 ± 1
1 (15)	37	96–121; 16	132–148; 17	48	42	1100	34	41 ± 4	76 ± 43
1 (20)	34	93–118; 18	131–147; 14	43	33	1300	190	26 ± 4	260 ± 30
1 (30)	24	87–120; 12	124–142; 14	36	26	710	310	30 ± 2	470 ± 40
1 (40)	14	82–111; 10	118–138; 12	28	17	270	140	21 ± 3	150 ± 20
2 (10)	43	100–121; 17	136–151; 17	58	52	1600	38	42 ± 3	4 ± 2
2 (20)	31	94–110; 19	129–145; 16	45	36	1400	180	22 ± 3	440 ± 160
2 (30)	24	90–110; 14	125–143; 13	37	27	820	310	14 ± 6	540 ± 100
2 (40)	15	87–111; 7	123–138; 8	49, 30	23	580	260	—	—
4 (10)	49	107–140; 20	140–156; 18	54	49	1500	35	21 ± 7	31 ± 13
4 (15)	41	96–116; 30	143–151; 27	39	30	680	150	17 ± 0.4	180 ± 6
4 (20)	35	94–122; 28	142–149; 24	43	34	1300	170	9 ± 1	110 ± 20
4 (30)	25	88–110; 23	123–145; 20	36	—	320	49	—	—
4 (40)	21	83–103; 26	134–143; 23	33	—	360	60	—	—
ATBC (10)	46	98–125; 25	139–155; 25	52	46	2000	43	52 ± 3	5 ± 3
ATBC (15)	37	95–119; 30	135–154; 26	47	38	1500	100	26 ± 0.4	220 ± 14
ATBC (20)	30	87–100; 28	141–152; 27	42	32	840	120	27 ± 1	300 ± 57

Plasticizer 1 facilitated the crystallization of PLA, and the cold crystallization temperature (*T*_cc_) signals were detected in the second heating DSC traces of the blends (Fig. 1a).^39,60,61^ In contrast, the *T*_cc_ signal was negligibly observed in neat PLA (Natureworks 2003D),^48,60^ even though some literature reports have indicated its presence.^32,34,49,50^ As the amount of 1 increased (10, 20, 30, and 40 phr), the *T*_cc_ signals gradually shifted to lower temperatures (103–125, 93–118, 87–120, and 82–111 °C, respectively), while the heat of crystallization remained almost constant (Δ*H*, 12–18 J g^−1^). This shift can be attributed to the increased chain mobility at higher plasticizer content or the plasticizer's role as a nucleating agent.^61^ Due to the occurrence of cold crystallization, the melting temperature (*T*_m_) signals were observed in the blended PLAs with the same intensity as the *T*_cc_ signals, whereas the *T*_m_ signal was very weak in neat PLA. The *T*_m_ signals also gradually shifted to lower temperatures compared to neat PLA (148–156 °C), reaching 140–153, 131–147, 124–142, and 118–138 °C with increasing amounts of 1 (10, 20, 30, and 40 phr, respectively). This shift is a common behavior observed in plasticized semicrystalline polymers, and it can be attributed to the presence of a less perfect crystalline phase resulting from higher levels of plasticizer content.^33,60^ For the blend containing 10 and 15 phr of 1, a single melting signal was observed, while two melting signals were observed for blends containing 20, 30, and 40 phr of 1. This behavior is commonly observed in PLA blends and is attributed to cold crystallization. The higher temperature signal corresponds to the α-form lamellar crystals, whereas the lower temperature signals correspond to the less perfect α′-form crystals.^62,63^ Similar trends were also observed for PLA/2 and PLA/4 blends in terms of *T*_cc_ and *T*_m_ (Table 1 and Fig. S7 and S8†).

**Fig. 2:**
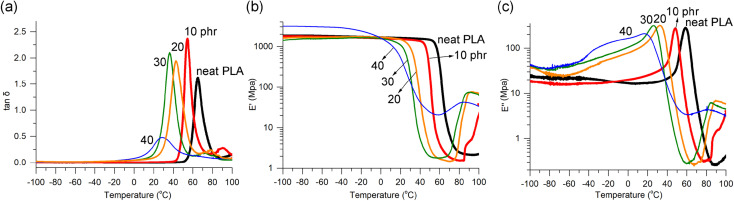
Temperature dependence of (a) tan *δ*, (b) storage modulus (E′), (c) loss modulus (E′′) curves from DMA runs illustrated for PLA/1 blends compared to neat PLA.

The homogeneity of plasticizer distribution was further demonstrated through dynamic mechanical analysis (DMA), a highly sensitive technique for detecting phase separation in blends. The blends containing 10, 20, and 30 phr exhibited fairly narrow and unimodal loss factor (tan *δ*) curves, indicating an even distribution of 1 within the PLA matrix (Fig. 2a). However, PLA/1_40 phr_ showed a broad and weak, yet still distinguishable tan *δ* signal. In cases of uneven distribution, wide signals with fluctuating multiple peaks or shoulders are typically observed.^36^ For well-known plasticizers, such as triethyl citrate and ATBC, prominent tan *δ* curves were observed up to 17.5 phr content but became very broad at 25 phr or higher content.^64^ The peaks of tan *δ* signals, also known as α-relaxation transition temperature (*T*_α_), indicated the *T*_g_ values, which gradually decreased with increasing amounts of plasticizer 1, as was observed in DSC studies. The *T*_α_ values were found to be approximately 10 °C higher than the *T*_g_ values measured using DSC.

The storage modulus (E′) curves of the blends containing up to 30 phr of 1 exhibited similar features to neat PLA. They showed a plateau line in the glassy state, followed by a sharp drop due to glass transition, and a subsequent increase due to cold crystallization (Fig. 2b). The temperature at which the E′ drop occurred (*i.e.*, *T*_g_) gradually decreased, consistent with the findings from DSC studies. The levels of the plateau lines (*i.e.*, E′ values) of the blends remained almost unchanged after blending with 1 (10, 20, and 30 phr), which is in contrast to the findings reported for blends with acetylated oligolactide. In those blends, the E′ value decreased from 2.9 GPa in neat PLA to 0.1–1.3 GPa in the blends.^47^ The shape of the E′ curve for PLA/1_40 phr_ deviated somewhat from the features observed in neat PLA and the blends containing 10, 20, and 30 phr. It did not exhibit the sharp drop observed in the other cases, which aligns with the broad *T*_g_ signal observed in DSC and the broad and weak tan *δ* signal. Similar deviations were reported to be observed in the blends with ATBC when the plasticizer contents were 25 phr or higher, although the E′ curve feature looked normal up to 20 phr contents of ATBC (Fig. S9†).^64^ However, a substantial reduction of the E′ value at the glassy plateau region was observed for PLA/ATBC_20 phr_, from 1.8 to 1.5 GPa in neat PLA to 0.6–0.9 GPa.

The loss modulus (E′′) curves of the blends containing 10, 20, and 30 phr exhibited the same features as neat PLA, showing distinct peaks that allowed for determining the *T*_g_ values, which were almost identical to the values obtained from DSC (Fig. 2c). In cases where miscibility between PLA and the plasticizer is poor, resulting in phase separation, signals related to the plasticizer are typically observed, especially at low-temperature regions.^34,44^ However, such signals were absent in the blends with 1. The shape of the E′′ curve for PLA/1_40 phr_ also deviated somewhat from the typical features. The DMA runs of PLA/2 blends showed similar features in tan *δ*, E′, and E′′ lines compared to PLA/1 blends, with the exception of the observation of two tan *δ* signals in PLA/2_40 phr_ (Fig. S10†). The DMA curves for the blends containing 20 phr or lower amounts of plasticizer 4 exhibited the expected normal features. However, significant deviations from the normal features were observed when the amounts of plasticizer were high at 30 phr and 40 phr (Fig. S11†). In the tan *δ* curve, shoulder signals were observed around −40 °C, in addition to the main peak signals at 36 and 33 °C, respectively. In the E′ curve, a gradual decrease was observed instead of the typical sharp drop around *T*_g_, and the E′′ curve exhibited non-linearity in the low-temperature region below 0 °C.

**Fig. 3:**
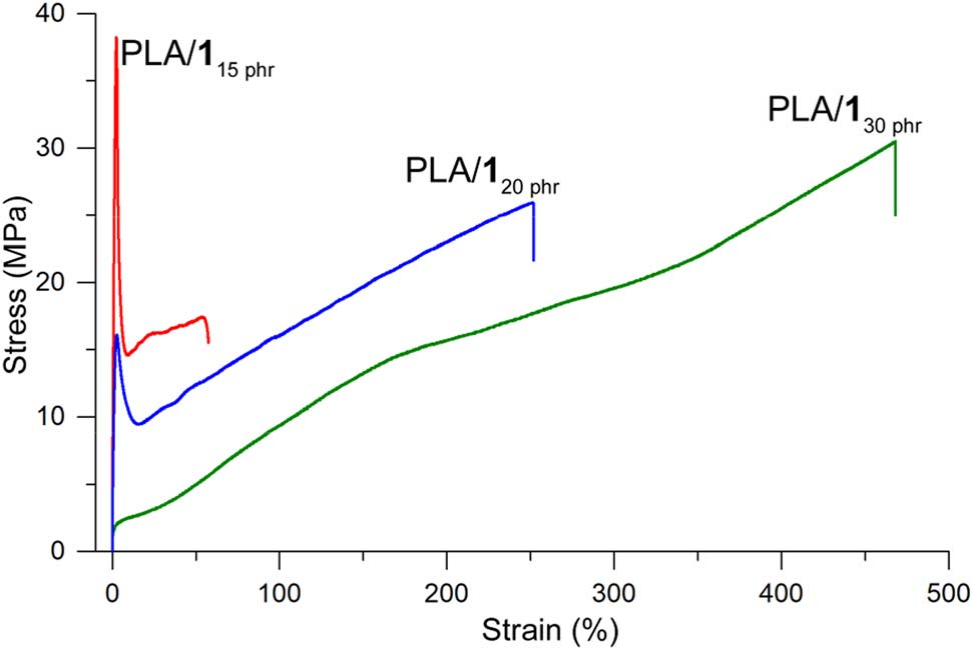
Stress–strain behavior for PLA/1 blends.

PLA/1_10 phr_ demonstrated brittleness similar to neat PLA. There was no significant improvement in elongation at break compared to neat PLA (3%), while the tensile strength marginally decreased (60 *vs.* 50 MPa) and Young's modulus slightly increased (2.1 *vs.* 2.4 GPa). These results suggest that the plasticizing effect was negligible at the small amount of 1 (10 phr). However, when the amount of 1 increased to 15 phr, the tensile curve exhibited ductile behavior. It showed an elastic region with a Young's modulus of 1.6 GPa, an upper yield point with an ultimate tensile strength of 41 ± 4 MPa, a lower yield point with a substantial rapid decrease in stress to 17 ± 2 MPa, strain hardening, and eventual breakage (Fig. 3). The elongation at break values were substantially increased compared to neat PLA, although they varied significantly depending on the specimens (76 ± 43%). PLA/1_20 phr_ displayed similar characteristics to PLA/1_15 phr_, but with considerably lower and non-uniform yield strengths (13 ± 7 MPa). The strain hardening effect was prominent, and elongation at break values were consistently high and uniform (260 ± 30%) with fairly high ultimate tensile strengths (26 ± 4 MPa). Finally, PLA/1_30 phr_ exhibited an elastomer-like tensile curve, without distinct yield points, but showing a simple strain hardening line from the origin to the breaking points. The tensile strength and strain at break were enhanced to 30 ± 2 MPa and 470 ± 40%, respectively, compared to PLA/1_20 phr_.

PLA/2_10 phr_ did not exhibit ductile properties, whereas PLA/2_20 phr_ and PLA/2_30 phr_ displayed ductile behavior with noticeable strains at break, albeit with notable fluctuations (440 ± 160 and 540 ± 100%, respectively; Fig. S12†). The significant fluctuations in strain at break indicated an uneven distribution of plasticizer 2 within the material. PLA/1_20 phr_ showed a distinct yield point, although it was non-uniform. In contrast, PLA/2_20 phr_ did not exhibit such a distinct yield point and instead showed a simple elastomer-like tensile curve, with a slightly reduced ultimate tensile strength (22 ± 3 MPa) compared to PLA/1_20 phr_ (26 ± 4 MPa). PLA/2_30 phr_ exhibited a tensile behavior similar to PLA/2_20 phr_, but with non-uniform and reduced ultimate tensile strength (14 ± 6 MPa) and strain (540 ± 100%) values. In accordance with the results in the DMA studies, PLA/4_30 phr_ and PLA/4_40 phr_ exhibited very weak mechanical strength, breaking at an early stage of low stress and strain. In contrast, blends with 10, 15, and 20 phr of 4 displayed ductile curves (Fig. S13†). However, the ultimate tensile strengths of these blends were substantially lower than those observed for the blends with 1. Specifically, the yield strength (ultimate tensile strength) of PLA/4_10 phr_ was non-uniform and low at 21 ± 7 MPa compared to PLA/1_15 phr_ (41 ± 4 MPa). The tensile curve of PLA/4_15 phr_ displayed a similar trend to that of PLA/1_20 phr_, but with an inferior tensile strength at break and less strain hardening effect (17 ± 0.4 *vs.* 26 ± 4 MPa).

In the case of the representative plasticizer ATBC, a similar negligible plasticizing effect was observed at a low amount of 10 phr.^33^ However, PLA/ATBC_15 phr_ also exhibited ductile properties (Fig. S14†), although its yield strength was substantially weaker compared to PLA/1_15 phr_ (18 ± 6 *vs.* 41 ± 4 MPa), while the elongation at break was higher (220 ± 14 *vs.* 76 ± 43%). Contrary to the reported limitations of ATBC's ability to uniformly plasticize PLA at 25 phr or higher content,^64^ as indicated by the observation of blurred tan *δ* curves, our findings revealed the presence of ductile properties with high tensile strengths and elongations at break in both PLA/ATBC_30 phr_ and PLA/ATBC_40 phr_. Specifically, PLA/ATBC_30 phr_ exhibited tensile strengths of 27 ± 2 MPa and elongations at break of 290 ± 37%, while PLA/ATBC_40 phr_ displayed tensile strengths of 26 ± 6 MPa and elongations at break of 464 ± 53%.

**Fig. 4:**
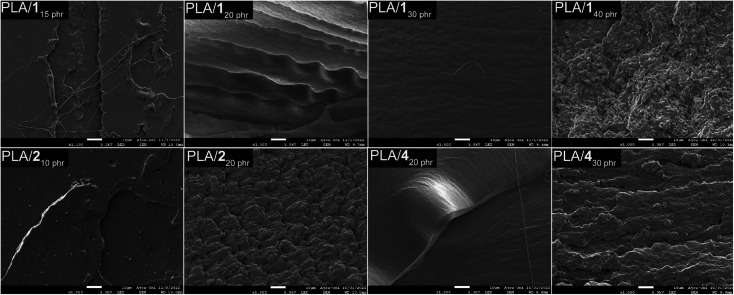
SEM images of the tensile fracture surfaces of PLA blends with 1, 2, and 4.

The miscibility of plasticizers with PLA was assessed through scanning electron microscopy (SEM) images (Fig. 4). Blends containing 30 phr or less of 1 exhibited smooth fracture surfaces, indicating good miscibility without phase separation. The observed SEM images aligned with the tensile properties that demonstrated ductile behavior with consistent strains at break (260 ± 30 and 470 ± 40%, respectively) and notable tensile strengths (26 ± 4 and 30 ± 2 MPa, respectively) for PLA/1_20 phr_ and PLA/1_30 phr_. However, PLA/1_40 phr_ displayed rough and irregular surfaces, suggesting phase separation. Due to phase separation, weak mechanical properties were observed in the PLA/1_40 phr_ blend. Conversely, SEM images revealed that 2 is unsuitable as a plasticizer due to its crystalline nature with a melting temperature of 80 °C. In fact, solid compounds are rarely employed as plasticizers. SEM images showed the presence of small dot-like particles (possibly crystals of 2) even in blends containing as little as 10 phr of 2 and exhibited highly rough surfaces in PLA/2_20 phr_ (Fig. S15†). The uneven distribution of plasticizer resulted in fluctuating tensile properties for PLA/2_20 phr_ and PLA/2_30 phr_. While both materials exhibited ductile behavior with relatively high average strains at break, they also displayed significant fluctuations in the strain at break (440 ± 160 and 540 ± 100%, respectively). Additionally, over time, plasticizer 2 migrated from the polymer matrix, forming solid particles on the blend's surface (“sweat-out” phenomenon). Blends containing 20 phr or less of 4 displayed smooth surfaces, while those with 30 phr or higher of 4 exhibited rough and irregular surfaces, indicative of phase separation (Fig. S16†). Notably, the detrimental “sweat-out” phenomenon was absent in samples showing smooth surfaces (*i.e.*, blends containing 30 phr or less of 1 and 20 phr or less of 4).

In gel permeation chromatography (GPC) studies, notable chain scissions were observed in the PLA chains during the blending process with 4. This led to a significant reduction in the weight-average molecular weight (*M*_w_). More specifically, the initial *M*_w_ of pristine PLA stood at 260 kDa with a dispersity (*Ð*) of 2.2. However, after undergoing identical thermal treatment without the addition of plasticizers, this value decreased to 180 kDa (*Ð* = 2.2). It's important to note that this reduction in *M*_w_ was consistently observed, resulting in *M*_w_ value of either 180 kDa or 170 kDa (*Ð* = 2.2), even when the thermal treatment was conducted under a N_2_ atmosphere with eliminating the possibility of water inclusion. This phenomenon, which is well-documented in the case of PLA, can be attributed to chain scission rather than hydrolysis.^65^

**Fig. 5:**
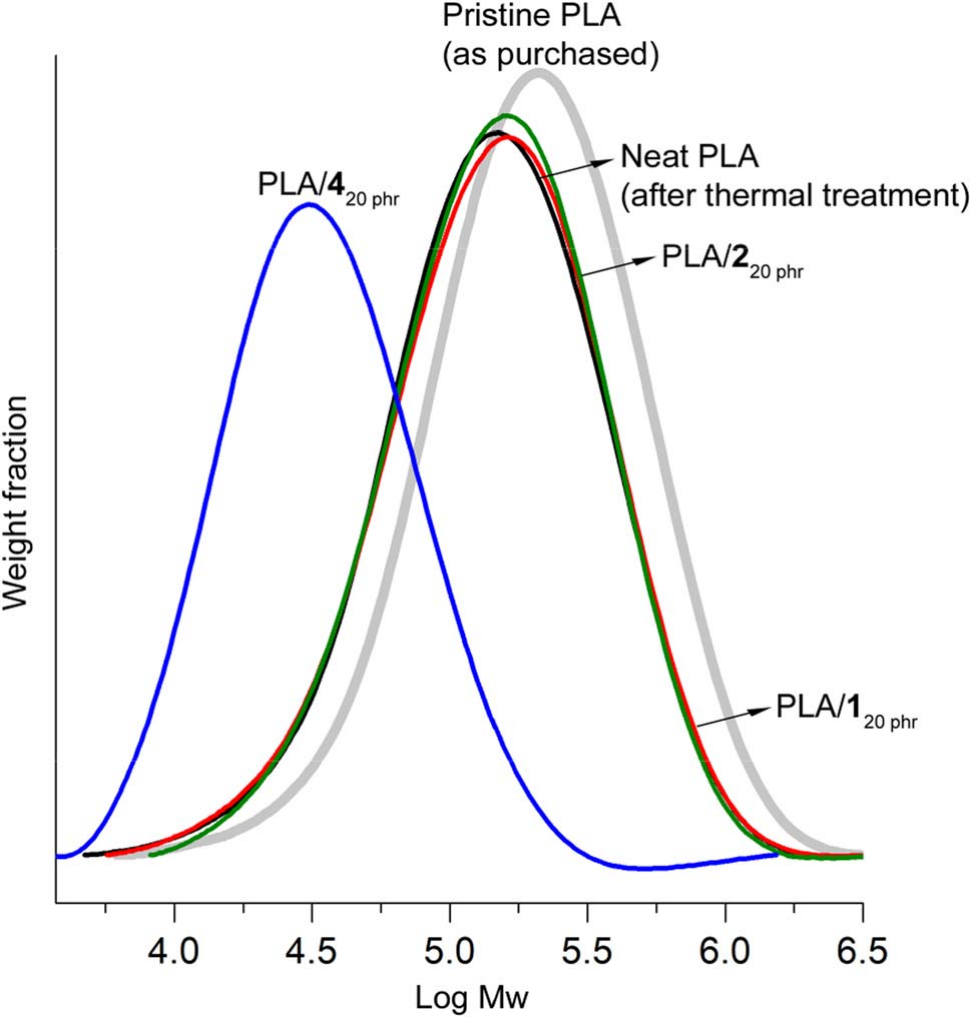
GPC curves of pristine purchased PLA, neat PLA post thermal treatment under blending conditions, PLA/1_20 phr_, PLA/2_20 phr_, and PLA/4_20 phr_.

In the case of PLA/4_20 phr_, the *M*_w_ value experienced a substantial decrease to 44 kDa (*Ð* = 2.0) (Fig. 5). The significant decrease in the *T*_g_ value from 60 to 35 °C can primarily be attributed to the plasticizer effect rather than the reduction in molecular weight. The *T*_g_ of PLA with a molecular weight (*M*_w_) of 44 kDa and a dispersity of 2.0 is estimated to be 58 °C using the modified Flory–Fox equation (*T*_g_ = *T*_g_^∞^ − 52/(*M*_*n*_ × *M*_w_)^0.5^ = 60 − 52/(22 × 44)^0.5^).^66^ This estimate is in close agreement with the reported *T*_g_ value of 55 °C found in the literature.^67^ Importantly, both of these values are not significantly lower than the *T*_g_ of high molecular weight PLA, which stands at 60 °C. This reduction in molecular weight contributed to the material's weak tensile properties, with a tensile strength of 9 MPa and a strain at break of 110%. Typically, commercial PLA grades are primarily synthesized through ring-opening polymerization of lactide using a tin(ii) catalyst.^68^ These catalyst residues might catalyze transesterification reactions between PLA and 4, which possesses an ester bond, consequently leading to molecular weight reduction. Interestingly, ATBC, another plasticizer with ester linkages, did not exhibit such molecular weight reduction observed in the blending with 4.^47^ In the case of PLA/ATBC_20 phr_, it maintained a *M*_w_ value of 200 kDa (*Ð* = 2.2). The ester bonds in ATBC may possess steric hindrance, which could impede transesterification reactions. In contrast, the ester bonds in 4 are more openly configured, making them highly susceptible to transesterification reactions.

Blending with 1 and 2, characterized by carbonate linkages exclusively, did not result in any discernible molecular weight reduction. The *M*_w_ values for PLA/1_20 phr_ and PLA/2_20 phr_ [190 kDa (*Ð* = 2.2) and 190 kDa (*Ð* = 2.1), respectively] closely paralleled that of neat PLA. The decrease in molecular weight during the blending process is a widely acknowledged concern, particularly evident when employing plasticizers containing alcohol groups.^38^ In literatures, a common method involved mixing PLA and plasticizers in chloroform at room temperature, followed by complete solvent evaporation to create blends. These blends were subsequently processed using a twin-screw extruder, typically operated at optimally minimized temperatures (*e.g.*, 130–150 °C) for a brief duration (*e.g.*, 20 min).^47^ In contrast, our research adopted a more practical blending approach that eliminated the use of a solvent and instead employed relatively rigorous conditions. We subjected the materials to a prolonged thermal treatment (2 h at 170 °C for melting) and an additional 30 min of stirring to ensure thorough blending and to assess the robustness of the blends. It is worth mentioning that even under these extremely rigorous blending conditions, the PLA/1 and PLA/2 blends, which were formulated with plasticizers consisting solely of carbonate bonds, remained structurally intact. This stands in stark contrast to the significant reduction in molecular weight observed during the formulation with 4, which possesses an ester bond. Generally, carbonate linkages exhibit greater resilience than ester linkages, whether in terms of hydrolysis or transesterification (transcarbonation) reactions.

**Fig. 6:**
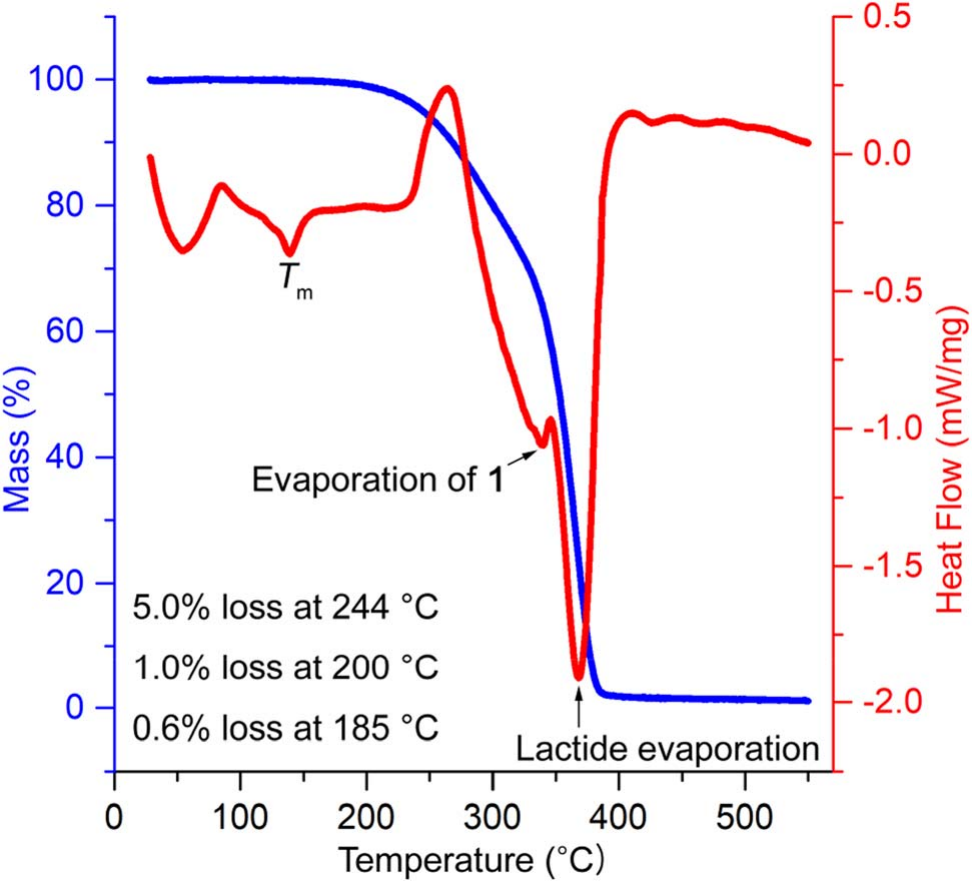
TGA/DSC analysis of PLA/1_30 phr_ blend.

The thermal stability of PLA/1 blends was evaluated using thermal gravimetry analysis (TGA) in combination with DSC. The temperatures corresponding to 5 wt% weight loss (*T*_d 5 wt%_) were found to be 277, 253, 244, and 244 °C for blends containing 10, 15, 20, and 30 phr of 1, respectively, and 206 °C for pure 1. These values were substantially lower than neat PLA (338 °C) (Fig. S17†) and were slightly higher than the acetyl-end-capped oligolactide plasticizer EtO[C(O)CH(Me)O]_4.5_C(O)Me (210–245 °C) reported previously.^47^ It is worth noting that the processing temperatures for PLA typically range from 185 to 250 °C, depending on the melting temperature (*T*_m_) of PLA, which ranges from 130 to 230 °C. The weight loss observed in PLA/1 blends was minimal, 0.56–0.76 and 1.0–1.5 wt%, at plausible processing temperatures of PLA/1 blends, *e.g.*, 185 and 200 °C, though the weight losses observed in pure 1 were significantly higher, reaching 2.4 and 4.0% at the same temperatures, respectively. In the DSC curves, two prominent overlapping signals were observed, peaking at 340–350 °C and 367–371 °C, along with small PLA *T*_m_ signals peaking at 152, 147, 143, and 139 °C, which gradually decreased with increasing amounts of plasticizer (Fig. S18†). These two major signals were reasonably attributed to the evaporation of 1 and lactide (formed by depolymerization in the sample holder), with reported boiling points of 305 and 286 °C, respectively. The onset temperatures of these signals were 270 °C or higher, indicating that they were sufficiently higher than the plausible processing temperatures of the blends, thereby alleviating concerns about the destruction or evaporation of 1 during thermal processing (Fig. 6).

**Fig. 7:**
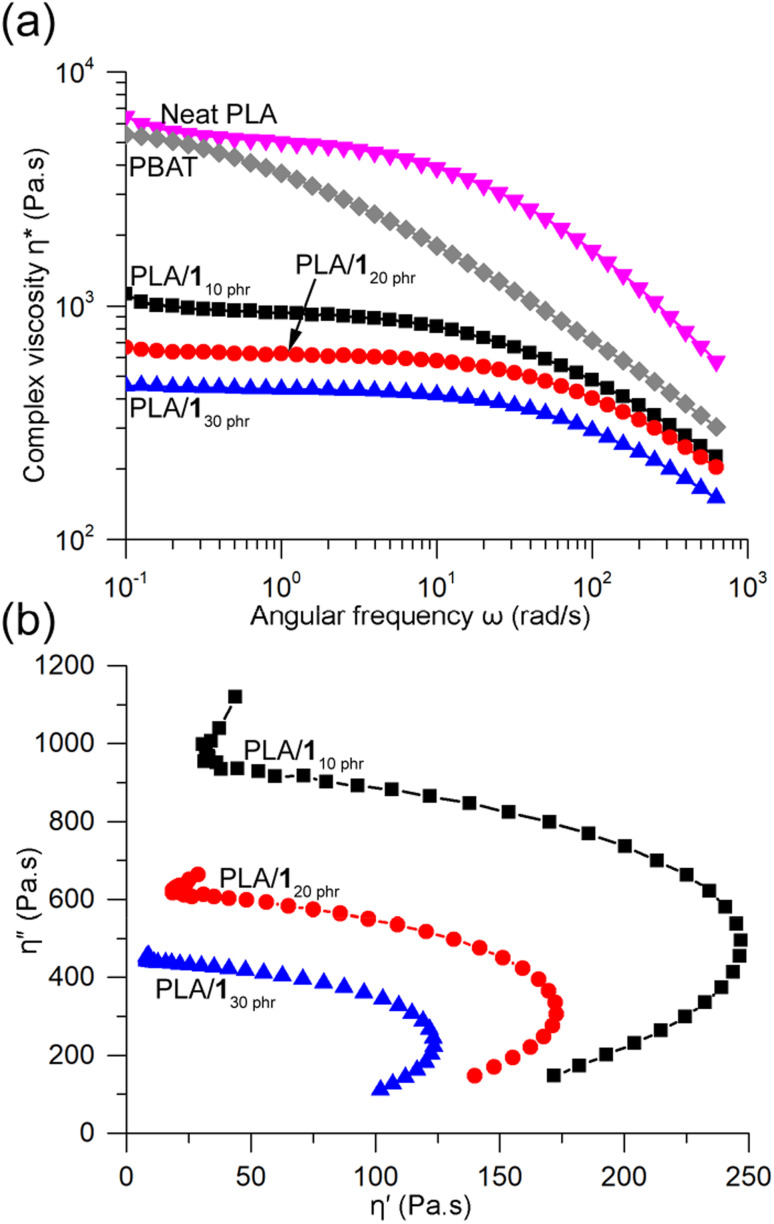
(a) Dynamic viscosities of PLA/1_10 phr_, PLA/1_20 phr_, and PLA/1_30 phr_ compared to neat PLA and PBAT measured using rotational rheometer at 170 °C. (b) Cole–Cole plots of PLA/1_10 phr_, PLA/1_20 phr_, and PLA/1_30 phr_.

The complex viscosities (η*) obtained from rotational rheometer measurements at 170 °C showed significant reductions upon blending, and the viscosity gradually decreased with increasing 1 content (Fig. 7a). For instance, at a low frequency of 0.1 rad s^−1^, the η* values for PLA/1_10 phr_, PLA/1_20 phr_, and PLA/1_30 phr_ were 1120, 660, and 450 Pa s, respectively. In contrast, neat PLA exhibited a substantially higher η* value of 6450 Pa s, comparable to that of another well-known biodegradable polyester, poly(butylene adipate-*co*-terephthalate) (PBAT), with *M*_w_ = 230 kDa (5400 Pa s). Both neat PLA and the blends displayed similar features in the η*-angular frequency curves, showing Newtonian plateau behavior at low frequencies and shear-thinning behavior at high frequencies.^61^ With an increase in plasticizer content, the Newtonian plateau lines extended to higher frequencies. In contrast, PBAT exhibited shear-thinning behavior across the entire frequency range, likely due to the broad dispersity of the commercial grade PBAT sample (*M*_w_/*M*_*n*_ = 4.4). Such shear-thinning behavior is beneficial in polymer processing techniques like blow molding and blown film extrusion.^69^ Certain PLA blends, such as the one containing 25 wt% glyceryl triacetate, were reported not to exhibit any shear-thinning behavior.^43^ PLA/1_10 phr_, PLA/1_20 phr_, and PLA/1_30 phr_ blends, as well as PBAT, displayed a viscous nature throughout the measured frequency range (*i.e.*, loss modulus G′′ > storage modulus G′) (Fig. S19†). In contrast, neat PLA exhibited a transition from viscous to elastic behavior at a critical frequency of 126 rad s^−1^.^50^ The Cole–Cole plot, representing the relationship between the real (*η*′) and imaginary (*η*′′) parts of η*, revealed semicircular lines for PLA/1_10 phr_, PLA/1_20 phr_, and PLA/1_30 phr_ blends, indicating good compatibility and phase homogeneity between PLA and 1 even in the melt state (Fig. 7b).^40^

**Fig. 8:**
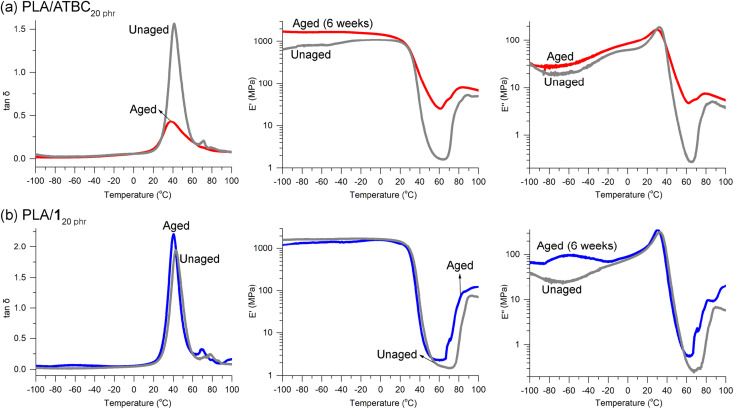
DMA curves for unaged and aged (6 weeks) specimens of (a) PLA/ATBC_20 phr_ and (b) PLA/1_20 phr_.

ATBC is widely recognized as one of the most efficient plasticizers for PLA due to its biobased origin, biodegradability, biocompatibility, and approval for food contact applications.^70^ However, concerns have been raised about the morphological stability of PLA when blended with tributyl citrate (TBC).^34^ The inclusion of plasticizer TBC leads to the crystallization of PLA chains even at room temperature, resulting in a reduction in the size of amorphous domains. Consequently, this phenomenon leads to a gradual opaqueness and deformation of the specimens over time. Remarkably, after aging for 6 weeks at room temperature, a previously transparent PLA/ATBC_20 phr_ specimen underwent a discernible transformation, becoming hazy. DMA revealed a significant weakening of the tan *δ* peak and pronounced alterations in the E′ and E′′ curves when compared to the unaged specimen (Fig. 8a). The decline in E′ and E′′ values across the *T*_g_ region was less abrupt and less dramatic than that observed in the unaged sample, implying a substantial crystallization process occurring during the 6 week storage period at room temperature. Crystallization due to aging was also evident in the wide-angle X-ray diffraction (WAXD) patterns. Initially, for PLA/ATBC_20 phr_, only broad signals were present at 2*θ* = 10–25°. Nevertheless, following the aging process, a distinct and sharp signal at 2*θ* = 16.5° emerged (Fig. S20a†), aligning with a prominent signal observed in PLA crystals. This signal can be attributed to the reflection from the (110/200) plane of the α′ or α crystal form of PLA.^61^

Crystallization in the specimens has been previously reported to result in a degradation of the tensile properties of PLA.^71^ In our observations, the tensile strength of neat PLA was found to decrease from 60 ± 2 to 50 ± 9 MPa when the specimens were annealed at 110 °C for 1 h to induce crystallization. This degradation in properties was similarly noted in the case of PLA/1_20 phr_. Notably, both the tensile strength and elongation at break decreased from 26 ± 4 to 21 ± 2 MPa and from 260 ± 30 to 10 ± 2%, respectively, after annealing under the same conditions. Encouragingly, the DMA curves of the PLA/1_20 phr_ specimen remained remarkably unchanged (Fig. 8b), and the specimen maintained its transparency throughout the 6 week aging period. This outcome suggests the absence of crystallization in the presence of plasticizer 1. Furthermore, in the WAXD curves of both unaged and aged specimens of PLA/1_20 phr_, only broad signals were detected (Fig. S20b†), in contrast to appearance of the clear and sharp signal in the aged PLA/ATBC_20 phr_. This absence of a sharp and distinct signal suggests that crystallization did not take place during the aging process.

### Biodegradability studies

**Fig. 9:**
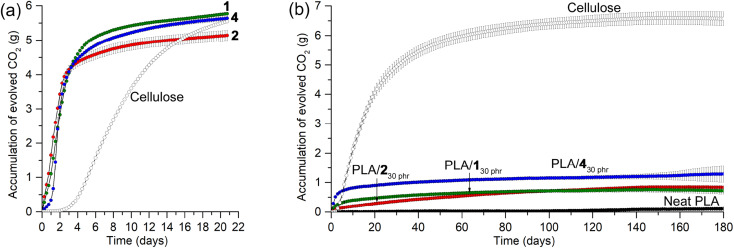
Biodegradability assessments of (a) organic carbonates and (b) their blends monitored by measuring evolved CO_2_ over time in a respirometer set to 25 °C and 50–55% water content with continuous air flow.

Prior to investigating the biodegradabilities of PLA/1_30 phr_, PLA/2_30 phr_, and PLA/4_30 phr_ blends, we first examined the biodegradabilities of organic carbonates 1, 2, and 4, individually, using the standard method and soil conditions in an ECHO^®^ respirometer set at 25 °C and 50–55% water content [ISO 17556 (2019)]. The evolved CO_2_ amount was continuously monitored under an air flow for 1, 2, and 4, compared to microcrystalline cellulose. Surprisingly, all tested organic carbonates exhibited very rapid biodegradability in soil at 25 °C, with almost complete consumption within a week, showing higher CO_2_-evolution rates than microcrystalline cellulose (Fig. 9a). The total evolved CO_2_ amounts in 2 weeks corresponded to 71, 69, and 68% of the CO_2_ amounts estimated for the full conversions of the carbons in 1, 2, and 4 to CO_2_ gas, respectively. It is worth noting that even in cases of complete biodegradation, not all carbon may evolve into CO_2_; a fraction could be assimilated into microbial biomass. Similarly, only 68% of the estimated CO_2_ was detected in the case of microcrystalline cellulose, suggesting that a portion of the carbon might have been assimilated into microbial biomass rather than being fully evolved into CO_2_. Glycerol carbonate 3 and propylene carbonate also exhibited similar rapid biodegradability (Fig. S21†). After unexpectedly observing rapid biodegradability of the organic carbonates, we assessed the stability of 2 in water, as it is fairly soluble, by monitoring its ^1^H NMR spectra in D_2_O over a period of 2 months (Fig. S22†). In 10 days, most of the signals corresponding to 2 remained intact, with the appearance of a set of small intensity signals assigned to glycerol carbonate 3 (5 mol%). The intensity of the signals corresponding to 3 increased over time without the formation of other signals, reaching 11 mol% in 30 days and 18 mol% in 60 days. The observed stability of organic carbonates in water implies that their rapid biodegradability is attributable to the collective effects of various soil constituents, including microbial communities.

PLA is non-biodegradable under ambient conditions in soil, and the CO_2_ evolution was minimal in the respirometer conditions at 25 °C and 50–55% water content, even after 180 days (1.1% of the estimated CO_2_ evolution; Fig. 9b). The PLA/1_30 phr_, PLA/2_30 phr_, and PLA/4_30 phr_ blends exhibited slightly higher rates of CO_2_ evolution that neat PLA, but this could be attributed to the biodegradation of the plasticizers rather than substantial PLA degradation. The total evolved CO_2_ amounts in 180 days corresponded to 39, 48, and 68% of those estimated for the complete conversion of the carbons in plasticizers 1, 2, and 4 to CO_2_ gas, respectively. After incubation in the respirometer for 180 days, PLAs in soils were extracted with CH_2_Cl_2_, and pure PLA was isolated by filtration of the CH_2_Cl_2_ solution through a short pad of silica gel, as confirmed by the analysis of ^1^H NMR spectra. The *M*_w_ values were 200 kDa (*Ð* = 2.1), 95 kDa (*Ð* = 2.0), and 130 kDa (*Ð* = 2.1) for PLAs extracted from soils incubated with pristine PLA, PLA/1_30 phr_, and PLA/2_30 phr_, respectively. The *M*_w_ values were only marginally reduced, indicating that PLAs, both individually and in blends, remained largely intact. This observation is consistent with PLA's known resistance to degradation in ambient conditions, whereas it is completely degraded under harsh industrial composting conditions with higher temperature of 60 °C and abundant microbiomes.

## Experimental

### General remarks

Reagents of glycerol, DEC, acetic anhydride, lithium *tert*-butoxide, Amberlyst^®^ 15 in hydrogen form (4.7 mmol-H^+^/g), cyclohexane, hexane, and anhydrous grade DMC were procured from Merck. ATBC was acquired from TCI (Tokyo Chemical Industry) and used as received, with precautions taken to prevent moisture exposure. PLA (Grade 2003D) was supplied by NatureWorks; prior to use, it was dried for 24 h in a vacuum oven at 70 °C. ^1^H NMR (600 MHz) and ^13^C NMR (150 MHz) spectra were recorded using a JEOL ECZ600 spectrometer. Thermal transition temperatures (*T*_g_, *T*_cc_, and *T*_m_) along with enthalpy of melting (Δ*H*) were determined *via* a DSC 200F3 Maia^®^ differential scanning calorimeter (DSC) from NETZSCH. Measurements were taken during the second heating cycle at a rate of 10 °C min^−1^. DMA was performed using a TA Instruments TA Q800 dynamic mechanical analyzer. Single cantilever mode was employed, heating from −100 to 100 °C at a constant rate of 3 °C min^−1^. The deformation amplitude was set at 15 μm, and frequency at 1 Hz. Tensile tests followed the KSM527-2 type 5A standard test method, utilizing a Qmesys QM100T universal testing machine. Tests were conducted at a drawing rate of 50 mm min^−1^ with a gauge length of 25 mm. Morphological analysis was carried out using a field emission SEM (JSM-7900F, JEOL) at an accelerating voltage of 2.0–3.0 kV. GPC data were acquired at 40 °C, using chloroform as an eluent and Shodex GPC HK-400 series with polystyrene standards. TGA/DSC experiments were performed using a NETZSCH model STA449F3 Jupiter^®^ within the temperature range of 35–700 °C. The heating rate was set at 10 °C min^−1^ under a N_2_ flow rate of 50 mL min^−1^. Dynamic shear melt rheological data were obtained using a TA Instruments DHR10 rotational rheometer at an angular frequency of 0.1–628 rad s^−1^ and a strain of 1% at 170 °C.

### 4-Ethoxycarbonyloximethyl-[1,3]dioxolan-2-one (1)

Glycerol (50.0 g, 0.543 mol), DEC (449 g, 3.80 mol), ^*t*^BuOLi (0.435 g, 5.43 mmol, 1.0 mol%), and cyclohexane (70 mL) were combined in a one-necked flask. The flask was fitted with a Dean–Stark apparatus, with the trap containing two layers of distilled water (400 mL) and cyclohexane (130 mL), connected to a manifold equipped with vacuum and N_2_ gas lines (Scheme 2c). After purging with N_2_ gas, the reaction mixture was placed in an oil bath set at 120–125 °C and stirred for 21 hours. Throughout the reaction, the resulting byproduct EtOH was continuously removed *via* the Dean–Stark apparatus. The volume of the aqueous phase gradually increased due to the absorption of the generated EtOH, and a portion of the aqueous phase was periodically discarded to prevent its flow into the reactor. To remove the base catalyst, ion exchange resin (Amberlyst^®^ 15 hydrogen form, 2.3 g, 11 mmol-H^+^), pre-washed with propylene carbonate and subsequently with DEC, was added. The solution was then filtered to eliminate the ion exchange resin. Excess DEC was removed by vacuum distillation under full vacuum at an oil bath temperature of 60 °C, followed by collecting the product as the oil bath temperature was raised to 150 °C (86.9 g, 81% yield). Although small impurity signals (primarily 1.5 mol% glycerol carbonate) were observed in the ^1^H and ^13^C NMR spectra of the collected portion (Fig. S1†), it was used without further purification for blending studies. ^1^H NMR (600 MHz, (CD_3_)_2_CO): *δ* 5.14 (m, H), 4.69 (dd, *J* = 9.0, 8.4 Hz, H), 4.47 (dd, *J* = 12.6, 3.0 Hz, H), 4.42 (dd, *J* = 9.0, 6.6 Hz, H), 4.39 (dd, *J* = 12.6, 4.8 Hz, H), 4.18 (q, *J* = 7.8 Hz, 2H), 1.26 (t, *J* = 7.8 Hz, CH_3_, 2H) ppm. ^13^C NMR (150 MHz, (CD_3_)_2_CO): 154.8, 74.4, 66.7, 66.0, 64.4, 13.8 ppm.

### 4-Methoxycarbonyloximethyl-[1,3]dioxolan-2-one (2)

The compound 2 was synthesized using the same procedure and conditions as 1. Glycerol (50.0 g, 0.543 mol), DMC (342 g, 3.80 mol) as a replacement for DEC, ^*t*^BuOLi (0.435 mg, 5.43 mmol, 1.0 mol%), hexane (200 mL), and water (400 mL) were utilized in the reaction. The reaction was conducted at a bath temperature of 85 °C for 28 hours. Upon completion of the reaction, while the reaction solution was still hot, ion exchange resin (Amberlyst^®^ 15 hydrogen form, 2.3 g, 11 mmol-H^+^) was added, followed by filtration after a 1 hour neutralization reaction. To remove some of the volatiles (hexane and DMC), the solution was subjected to rotary evaporation, resulting in a 180 g solution from which crystalline solids precipitated upon storage at ambient temperature for 12 hours (46.0 g, 48%). The purity of the isolated solids as 2 was confirmed by ^1^H and ^13^C NMR spectra (Fig. S2†). Additional yield of the product was obtained as second crops when the filtrate was stored overnight at ambient temperature after reducing its mass to 65 g using a rotary evaporator (14.9 g, 16%, total yield 64%). ^1^H NMR (600 MHz, (CD_3_)_2_CO): *δ* 5.13 (m, H), 4.69 (dd, *J* = 9.0, 8.4 Hz, H), 4.48 (dd, *J* = 12.6, 3.0 Hz, H), 4.42 (dd, *J* = 9.0, 6.6 Hz, H), 4.40 (dd, *J* = 12.6, 4.2 Hz, H), 3.77 (s, CH_3_, 3H) ppm. ^13^C NMR (150 MHz, (CD_3_)_2_CO): 155.9, 154.7, 74.5, 67.1, 66.2, 55.1 ppm.

### Glycerol carbonate (3)

Glycerol (156 g, 1.69 mol), DMC (458 g, 5.08 mol), and ^*t*^BuOLi (0.678 g, 8.47 mmol, 0.5 mol%) were combined in a one-neck flask (1 L). After purging with N_2_ gas, the reaction mixture was stirred at 80 °C for 1.67 hours. Analysis by ^1^H NMR of a sample confirmed the formation of the desired product in good yield. However, there was still a remaining amount of glycerol (6.5 mol%) as well as some side product 2 (5.0 mol%). Nonetheless, byproduct MeOH and excess DMC were removed by vacuum distillation, which was performed at a bath temperature of 30–35 °C with the receiver cooled by a dry ice/acetone bath. Encouragingly, most of the remaining glycerol and 2 were converted to the desired product during the distillation process, leaving behind 1.3 mol% of glycerol (Fig. S3†). Finally, ion exchange resin (Amberlyst^®^ 15 hydrogen form, 3.6 g, 17 mmol-H^+^) was added to the resulting product to remove the base catalyst. After stirring for 1 hour at ambient temperature, the resin was filtered off, yielding a colorless oil (198 g, 99% yield), which was used without further purification for blending studies. ^1^H NMR (600 MHz, (CD_3_)_2_CO): *δ* 4.87 (m, H), 4.57 (dd, *J* = 8.4, 7.8 Hz, H), 4.43 (t, *J* = 5.4 Hz, –OH), 4.41 (dd, *J* = 8.4, 6.0 Hz, H), 3.88 (ddd, *J* = 12.6, 4.8, 3.0 Hz, H), 3.71 (ddd, *J* = 12.6, 6.0, 3.6 Hz, H), 3.77 (s, CH_3_, 3H) ppm. ^13^C NMR (150 MHz, (CD_3_)_2_CO): 155.7, 77.4, 66.2, 61.7 ppm.

### Glycerol 1-acetate 2,3-carbonate (4)

Glycerol carbonate (35.7 g, 302 mmol), acetic anhydride (37.0 g, 363 mmol), and ion exchange resin (Amberlyst^®^ 15 hydrogen form, 0.30 g, 1.4 mmol-H^+^) were added to a one-neck flask. The reaction mixture was then purged with N_2_ gas and stirred at 60 °C for 48 hours. After cooling, the ion exchange resin was removed by filtration, and the volatiles were eliminated using a rotary evaporator, giving a colorless oil (41.4 g, 86%). Analysis of the ^1^H NMR spectrum revealed that the isolated product was contaminated with 3.4 mol% of triacetyl glycerol (Fig. S4†). ^1^H NMR (600 MHz, (CD_3_)_2_CO): *δ* 5.10 (m, H), 4.67 (dd, *J* = 9.0, 8.4 Hz, H), 4.39 (dd, *J* = 9.0, 6.0 Hz, H), 4.38 (dd, *J* = 12.6, 3.0 Hz, H), 4.31 (dd, *J* = 12.6, 4.2 Hz, H), 2.06 (s, CH_3_, 3H) ppm. ^13^C NMR (150 MHz, (CD_3_)_2_CO): 170.4, 155.1, 74.7, 66.4, 63.7, 20.1 ppm.

### Polymer blending

With a restricted amount of in-house prepared plasticizer, we designed and fabricated a compact reactor (25 mL capacity) equipped with a mechanical stirrer for blending (Fig. S23†). PLA samples (18 g) were combined with the plasticizer (3.6 g, 20 phr) under a N_2_ atmosphere within the custom-made reactor and, subsequently, the reactor was immersed in an oil bath at 170 °C for a duration of 2 h, ensuring complete melting of the PLA (*T*_m_, 148–156 °C). Following the melting process, stirring was performed at 170 °C utilizing a custom-made screw-type blade, for a period of 30 min to ensure thorough blending. Upon completion of the blending procedure, the reactor was opened while still hot, and the blended polymers were extracted from the reactor using long-nose pliers. To preserve the quality and prevent moisture contact from the surrounding air, the obtained blended polymers were vacuum-sealed within polyethylene film using a countertop vacuum sealer (FoodSaver, FM5460-071).

### Biodegradable studies

To emulate the natural biodegradation conditions for PLAs, soil samples were collected from three geographically distinct sites: Mt. Gwanggyosan, Suwon, South Korea (37°20.1360 N 127°1.1640′ E); Ajou University's field campus in Suwon (37°17.1210 N 127°2.6710 E), South Korea; and flower beds in Hwaseong, South Korea (37°10.8030 N 126°58.8720 E), in compliance with ISO 17556 (2019). The collected soil was air-dried naturally at room temperature for 24 hours, homogenized, and then sieved through a 2 mm mesh using an AS 200 Control vibrating sieve (Retsch, Germany). The 2400 g portions of sieved soil were supplemented with mineral nutrients (0.2 g KH_2_PO_4_, 0.1 g MgSO_4_, 0.4 g NaNO_3_, 0.4 g CO(NH_2_)_2_, and 0.4 g NH_4_Cl per kg of soil) and then equitably combined. PLA blends (neat PLA, PLA/1_30 phr_, PLA/2_30 phr_, and PLA/4_30 phr_) were mechanically ground and sieved through a 2 mm mesh using a Type ZM 200 Ultra Centrifugal Mill (Retsch, Nordrhein-Westfalen). Microcrystalline cellulose (20 μm, Sigma-Aldrich, St. Louis, MO, USA) served as a positive control for biodegradability assays. In each reactor, 15 g of PLA blends or microcrystalline cellulose was uniformly blended with 800 g of soil and then aerobically incubated in a reactor (triplicates) at 25 °C in the dark using a 12-channel ECHO^®^ respirometer (ECHO Instruments, Slovenske Konjice, Slovenia). A blank soil sample (800 g) was also incubated to normalize CO_2_ evolution. Real-time monitoring of CO_2_ evolutions was enabled by an integrated near-infrared (NIR) detector in the ECHO^®^ Respiratory system, with a consistent air flow rate of 200 mL min^−1^ regulated by an integrated mass flow controller. Likewise, 15 g of organic carbonate plasticizers 1, 2, or 4 were each mixed with 800 g of soil and incubated under identical conditions.

## Conclusions

4-Ethoxycarbonyloximethyl-[1,3]dioxolan-2-one (1), synthesized using renewable glycerol and CO_2_-derived DEC (CO_2_ content in 1, 46 wt%), proves to be a highly effective plasticizer for PLA. The plasticization effect was evident through the gradual reduction in *T*_g_ as the concentration of plasticizer increased, following a linear relationship represented by the equation “*T*_g_ (°C) = 58.4 − 1.13 × [amount of 1 (phr)]”. DMA revealed PLA/1 blends exhibiting narrow and unimodal tan *δ* curves, along with distinctive E′ and E′′ curves, confirming excellent miscibility of PLA with up to 30 phr of 1. This was further supported by SEM images. The blending process endowed the PLA with ductile properties, leading to a remarkable increase in strain at break, up to 470% for PLA/1_30 phr_, however, inevitably with some reduction in tensile strength (25–40 MPa). Chain scission was not observed during the melt blending process at 170 °C, as indicated by GPC studies. TGA displayed minimal weight loss (∼1%) at a plausible processing temperature of 200 °C. The dynamic viscosity curves demonstrated that while the blends retained similar features to neat PLA, the complex viscosity was significantly reduced, highlighting their improved processability. The Cole–Cole plot suggested phase homogeneity in the melt state. Significantly, 1 outperformed conventional ATBC plasticizer in terms of morphological stability. Unlike PLA/ATBC blends, PLA/1 blends exhibited no cold crystallization over time at room temperature. Furthermore, all synthesized organic carbonates exhibited rapid biodegradability under ambient soil conditions at 25 °C within a week. However, even over an extended 6 month period under the same ambient conditions, the PLA in the blends remained predominantly undegraded.

## Author contributions

Hyeon Jeong Seo and Yeong Hyun Seo conducted the blending and collection of analysis data. Sang Uk Park and Hyun Ju Lee were responsible for preparing the plasticizers. Mi Ryu Lee and Jun Hyeong Park managed the data collection and analysis. Woo Yeon Cho conducted the biodegradation studies. Bun Yeoul Lee and Pyung Cheon Lee supervised the project and offered invaluable suggestions. All the authors read, edited, and approved the final manuscript.

## Conflicts of interest

The authors declare no conflict of interest.

## Supplementary Material

RA-014-D3RA08922C-s001
