# Supplementary material for: Glycerol-derived organic carbonates: environmentally friendly plasticizers for PLA
Source: RSC Adv. 2024 Feb 5;14(7):4702–16. doi: 10.1039/d3ra08922c (PMC10840682; doi:10.1039/d3ra08922c)

Electronic Supplementary Material (ESI) for RSC Advances.
This journal is © The Royal Society of Chemistry 2024

Supporting Information

Glycerol-Derived Organic Carbonates: Environmentally Friendly Plasticizer for PLA

Hyeon Jeong Seo‡, Yeong Hyun Seo‡, Sang Uk Park, Hyun Ju Lee, Mi Ryu Lee, Jun Hyeong Park, Woo Yeon Cho, Pyung Cheon Lee,* and Bun Yeoul Lee*

Department of Molecular Science and Technology, Ajou University, Suwon 16499, South Korea.

Correspondence to: Bun Yeoul Lee, bunyeoul@ajou.ac.kr and Pyung Cheon Lee, pclee@ajou.ac.kr

‡These authors contributed equally to this work.

Figure S1. ^{1}H and ^{13}C NMR spectra of **1**.

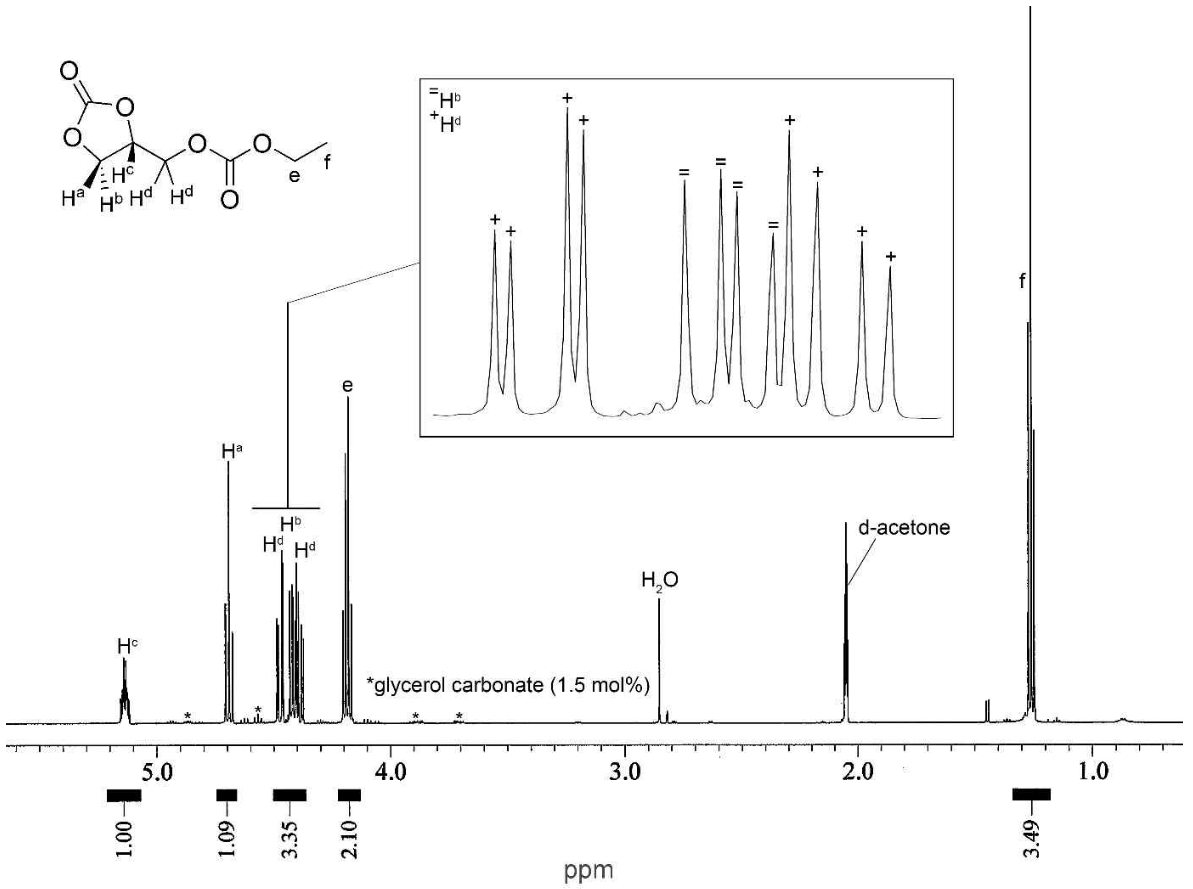

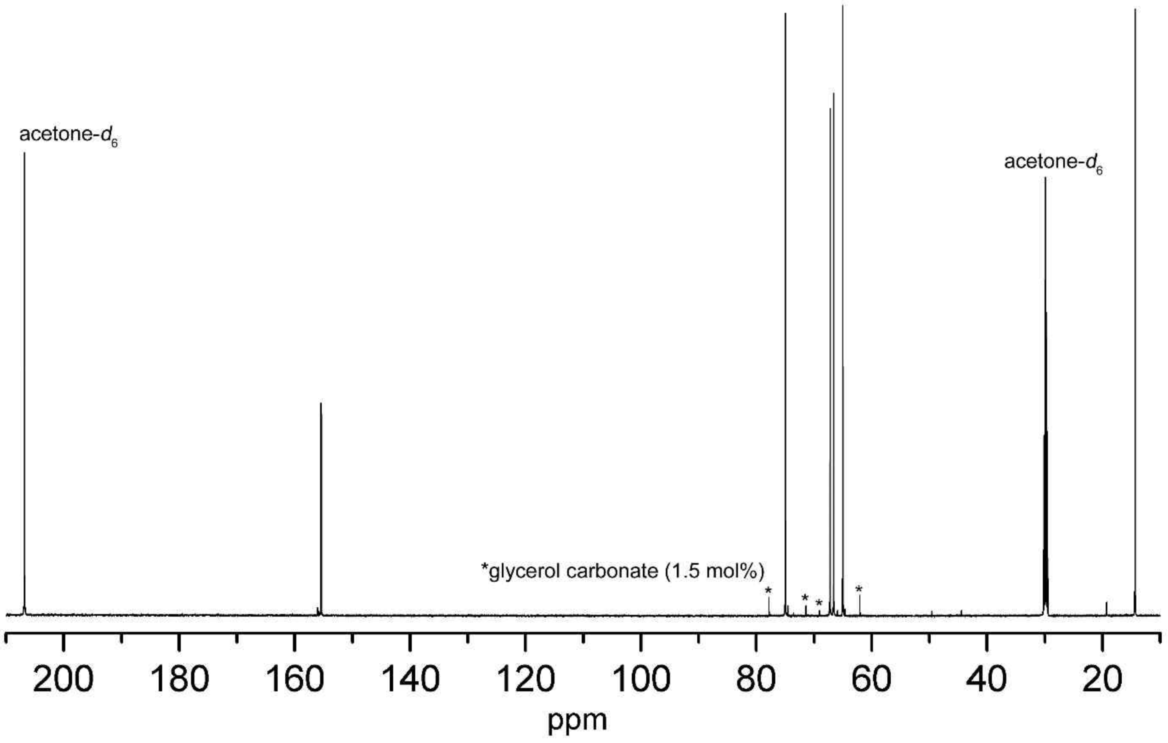

Figure S2. ^{1}H and ^{13}C NMR spectra of **2**.

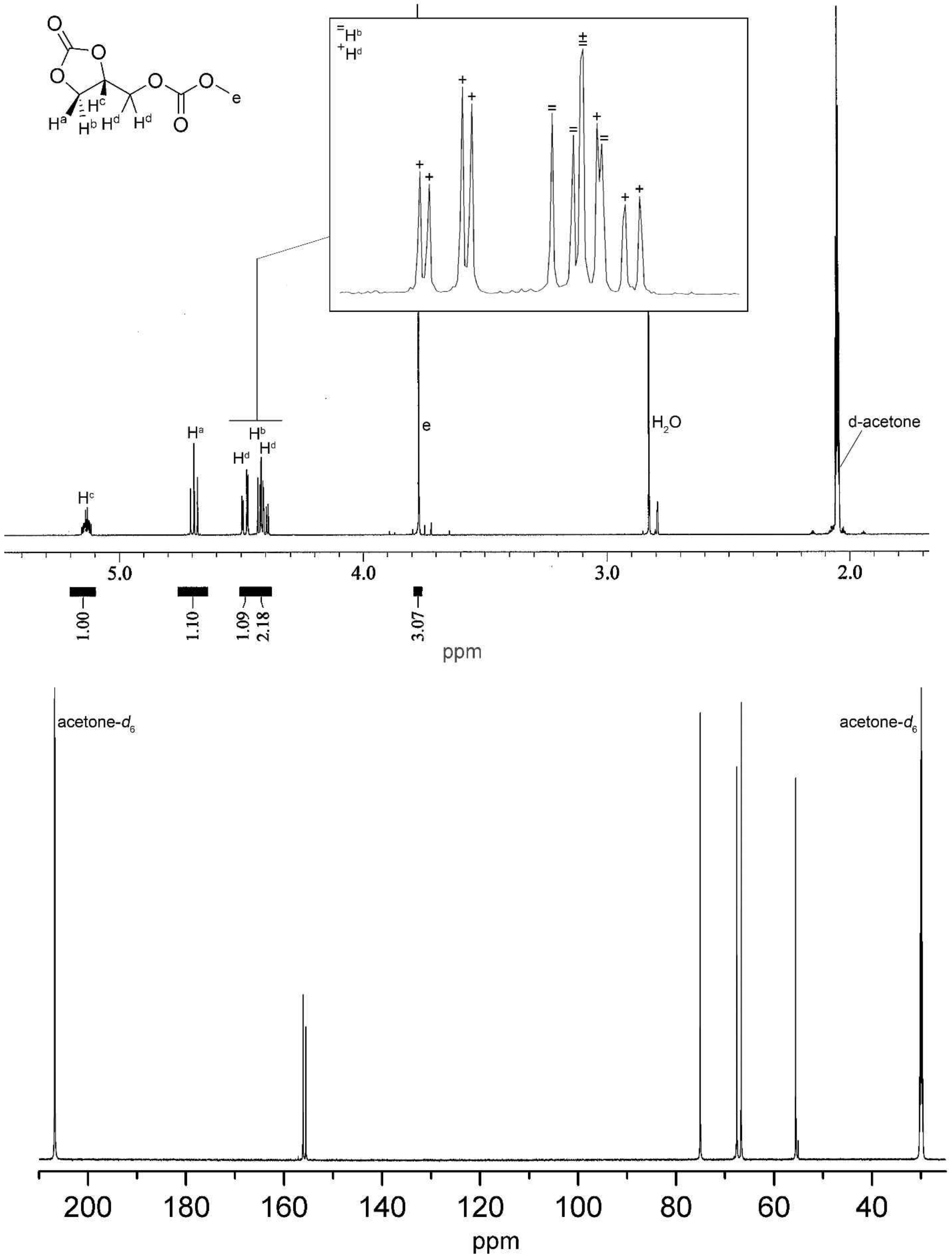

Figure S3. ^{1}H and ^{13}C NMR spectra of **3**.

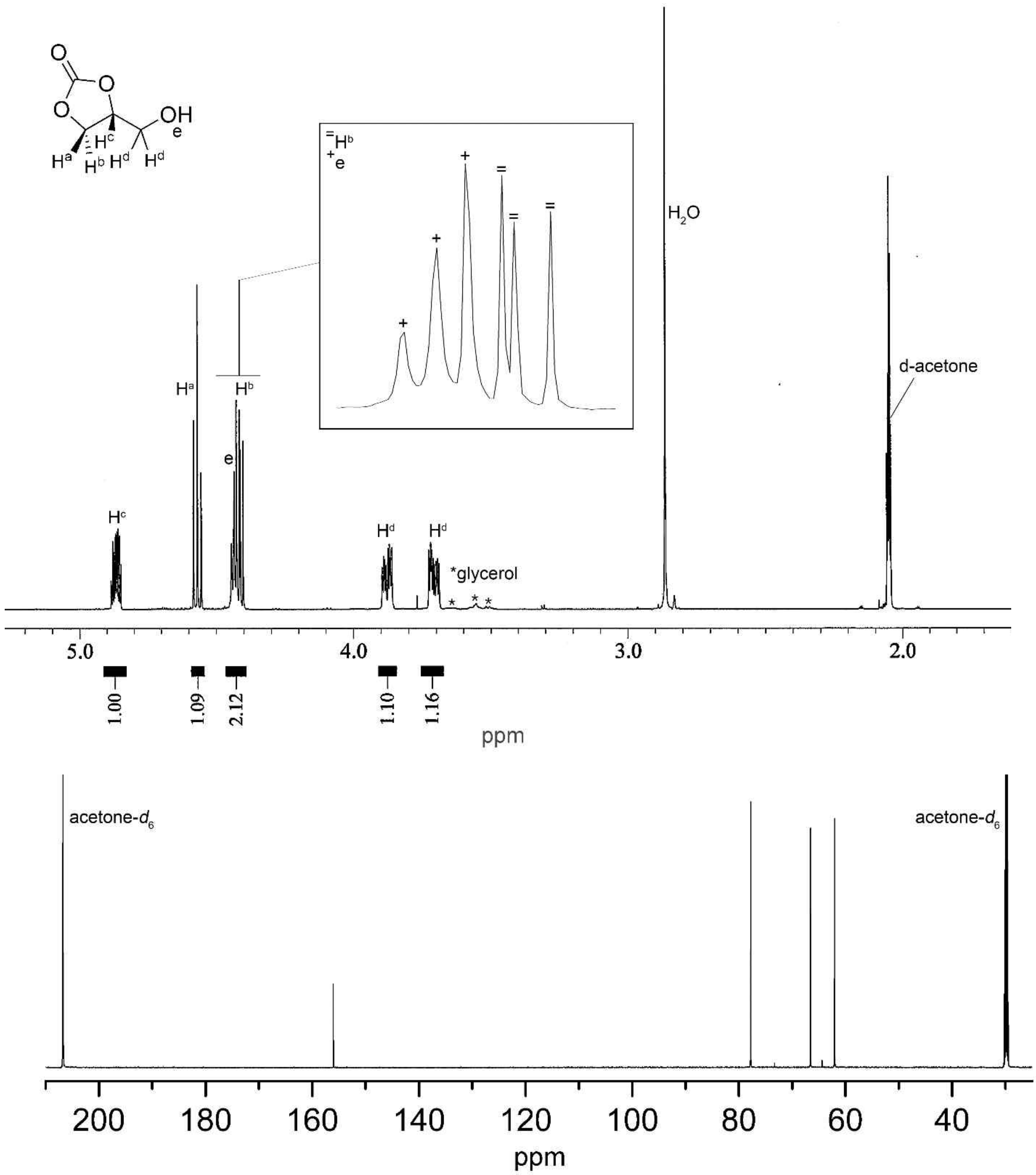

Figure S4. ^{1}H and ^{13}C NMR spectra of **4**.

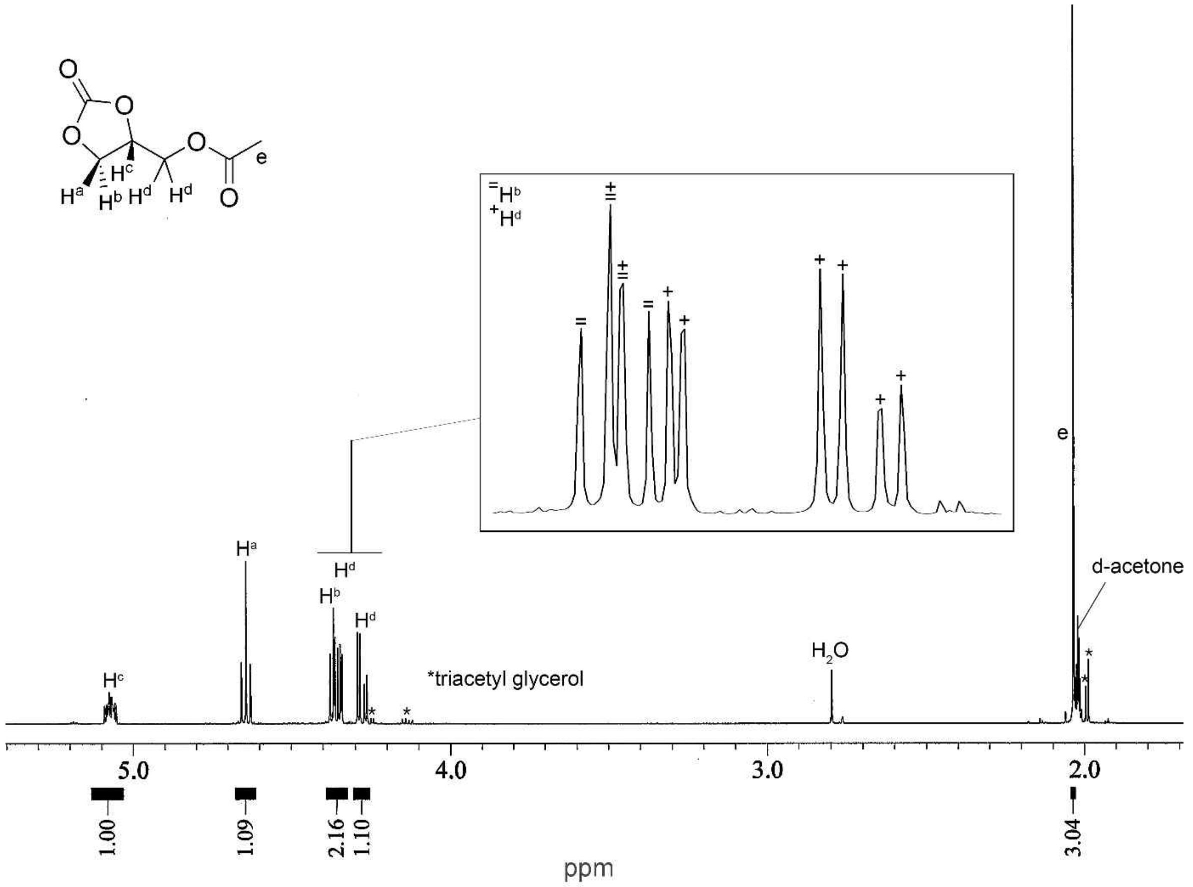

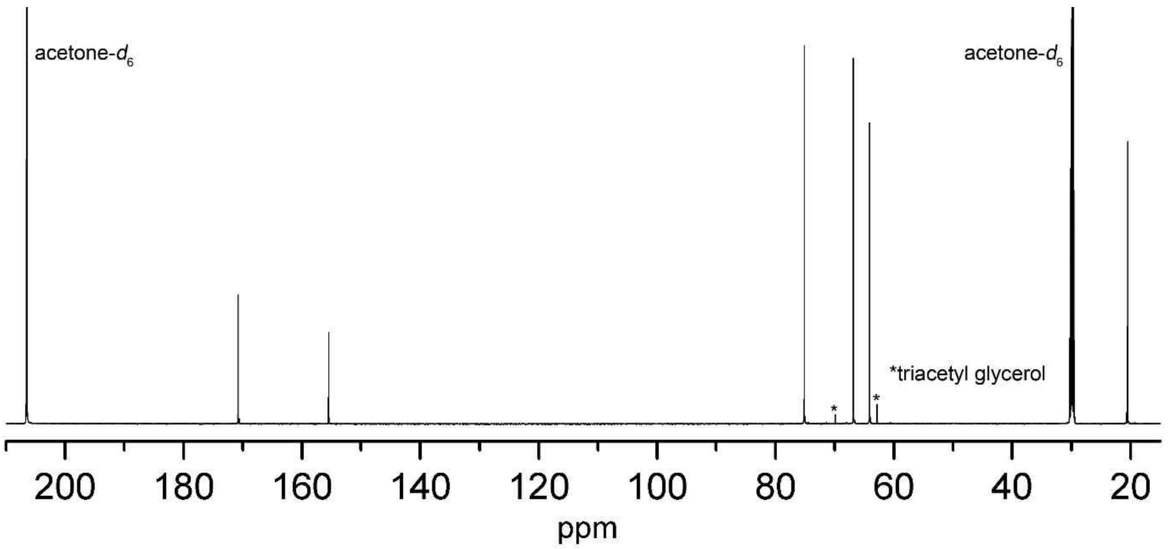

Figure S5. DSC traces of plasticizers **1**, **2**, **4**, and ATBC.

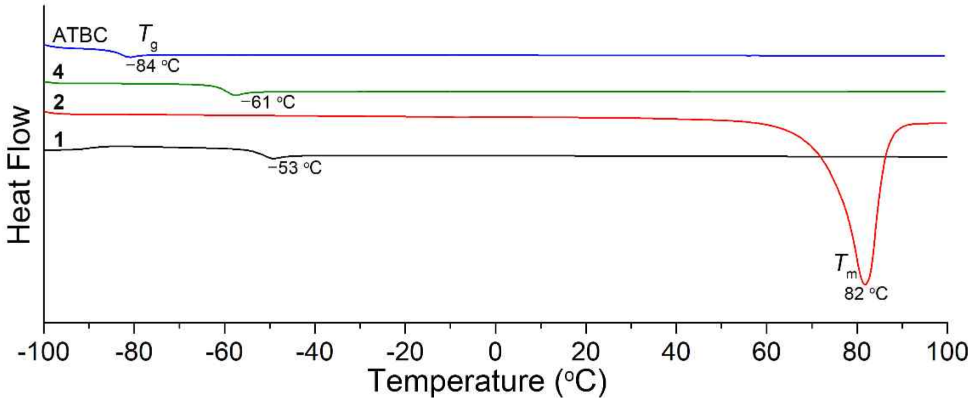

Figure S6. The second heating DSC traces of PLA/ATBC blends compared to neat PLA.

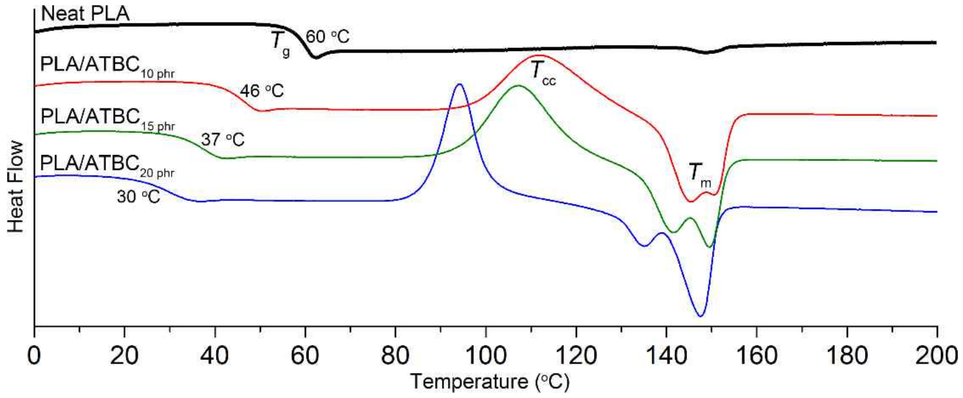

Figure S7. (a) The second heating DSC traces of PLA/**2** blends compared to neat PLA and (b) linear correlation between the content of **2** and T_g of the blends.

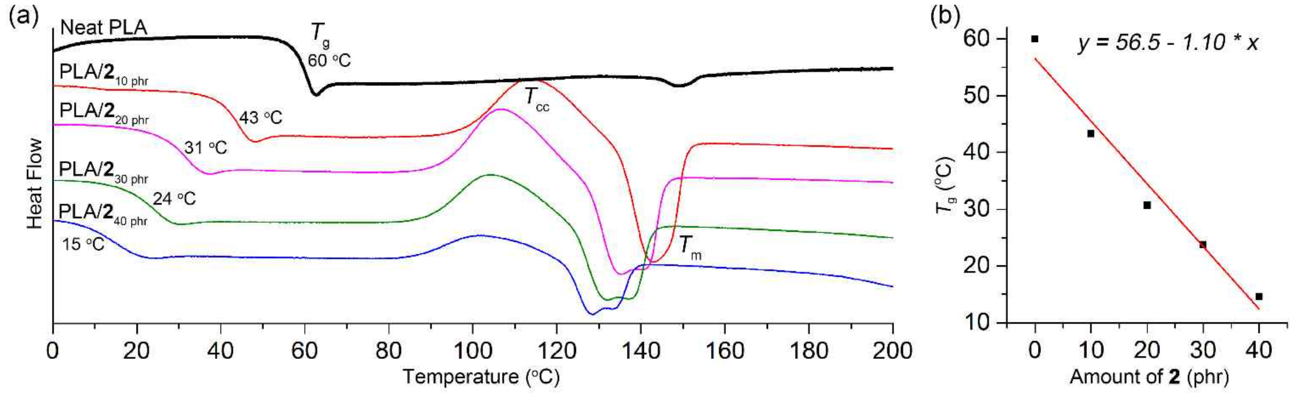

Figure S8. (a) The second heating DSC traces of PLA/**4** blends compared to neat PLA and (b) linear correlation between the content of **4** and T_g of the blends.

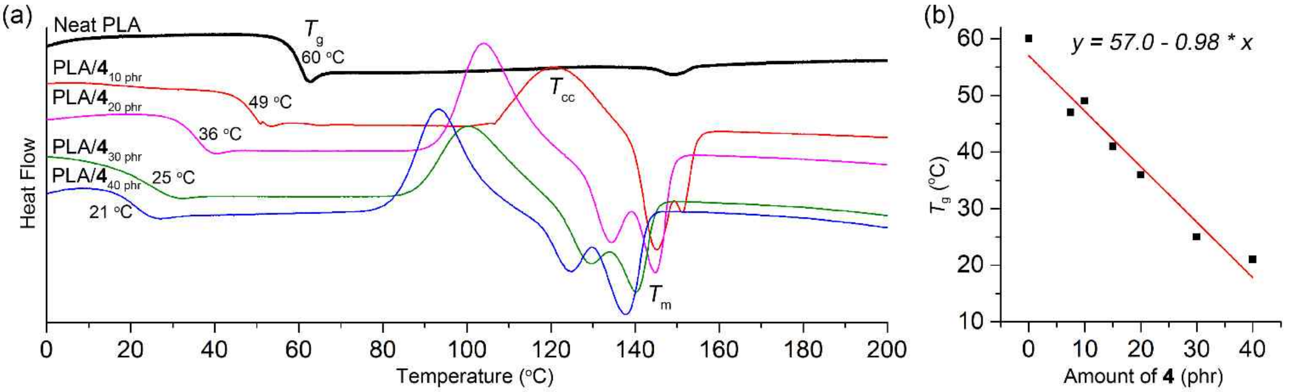

Figure S9. Temperature dependence of (a) tanδ, (b) storage modulus (E') and (c) loss modulus (E") curves from DMA runs illustrated for PLA/ATBC blends compared to neat PLA.

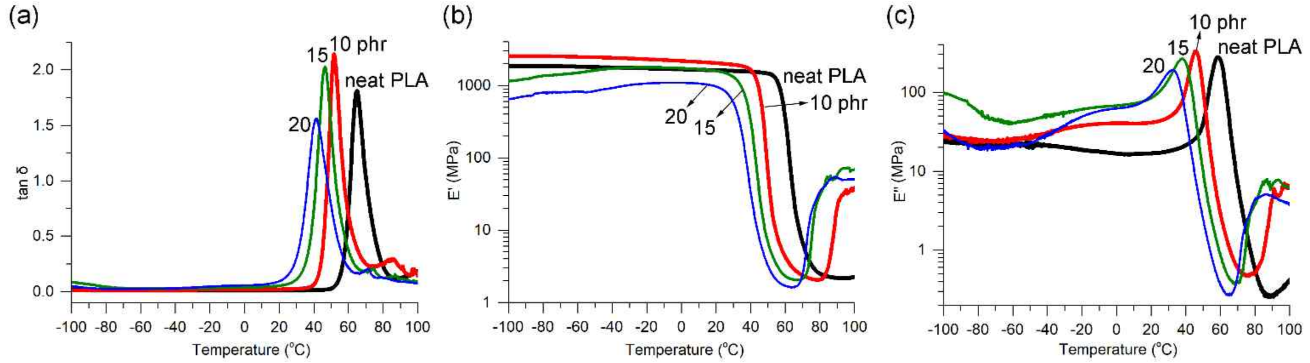

Figure S10. Temperature dependence of (a) tanδ, (b) storage modulus (E') and (c) loss modulus (E") curves from DMA runs illustrated for PLA/**2** blends compared to neat PLA.

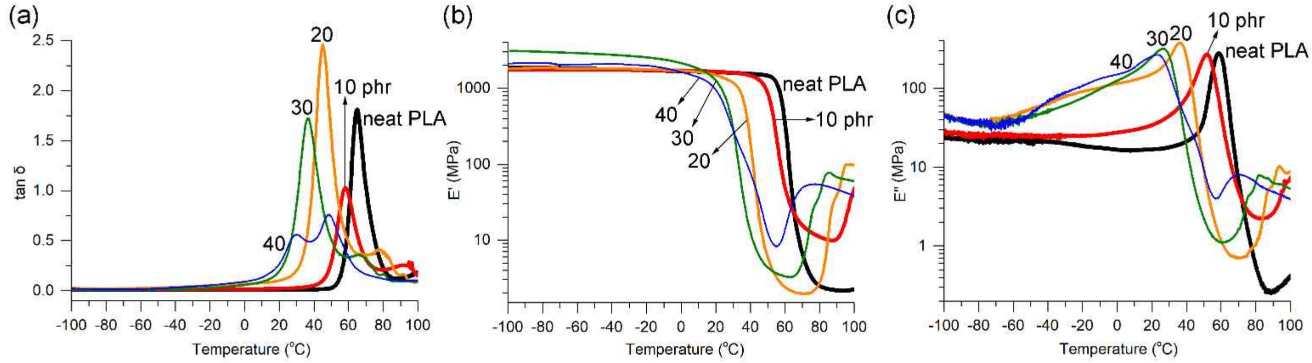

Figure S11. Temperature dependence of (a) tanδ, (b) storage modulus (E') and (c) loss modulus (E'') curves from DMA runs illustrated for PLA/**4** blends compared to neat PLA.

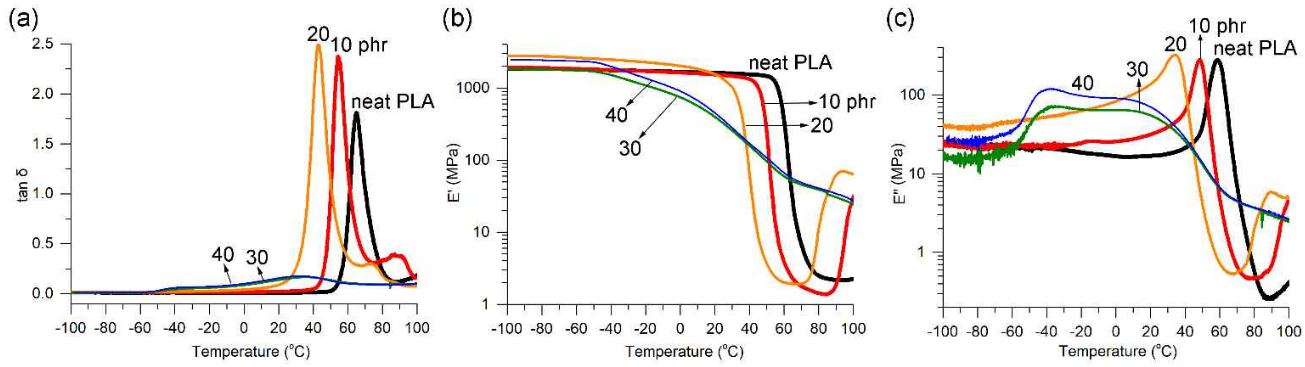

Figure S12. Stress-strain behavior for PLA/**2** blends compared to PLA/**1** blends.

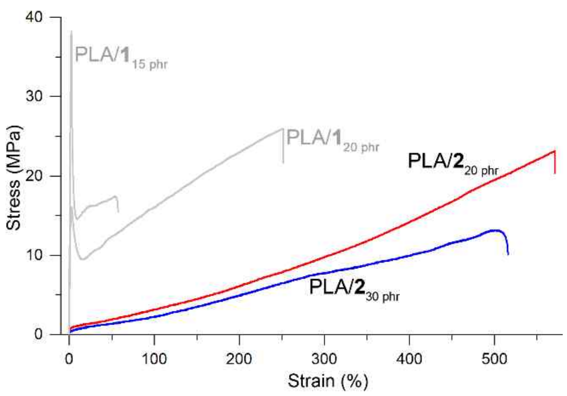

Figure S13. Stress-strain behavior for PLA/**4** blends compared to PLA/**1** blends.

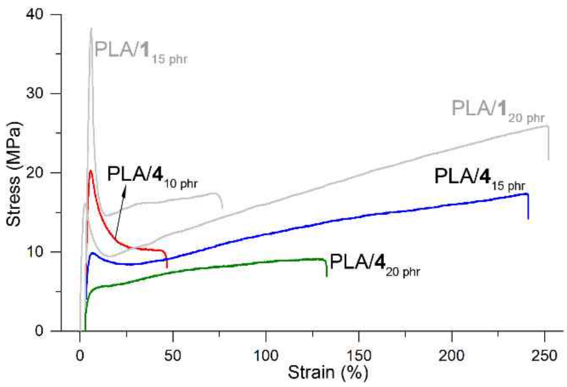

Figure S14. Stress-strain behavior for PLA/ATBC blends compared to PLA/**1** blends.

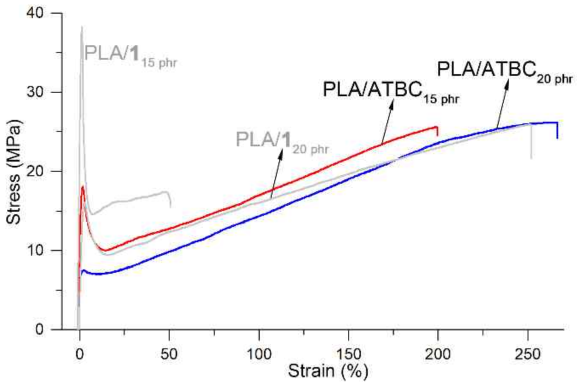

Figure S15. SEM images of the tensile fracture surfaces of PLA/**2** blends.

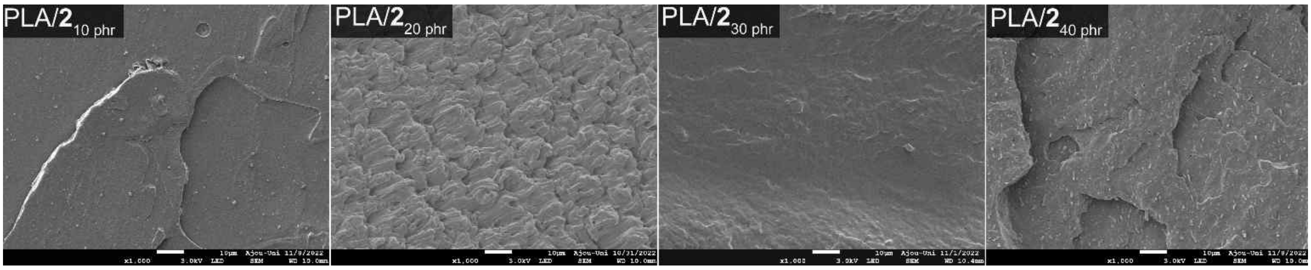

Figure S16. SEM images of the tensile fracture surfaces of PLA/**4** blends.

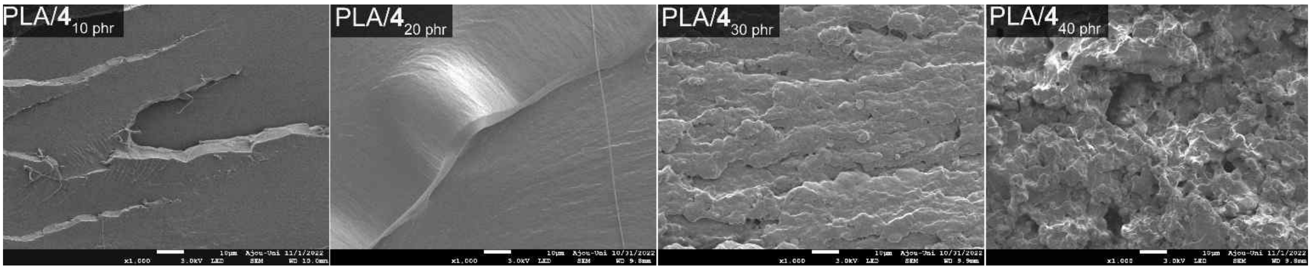

Figure S17. TGA curves of PLA/**1** blends.

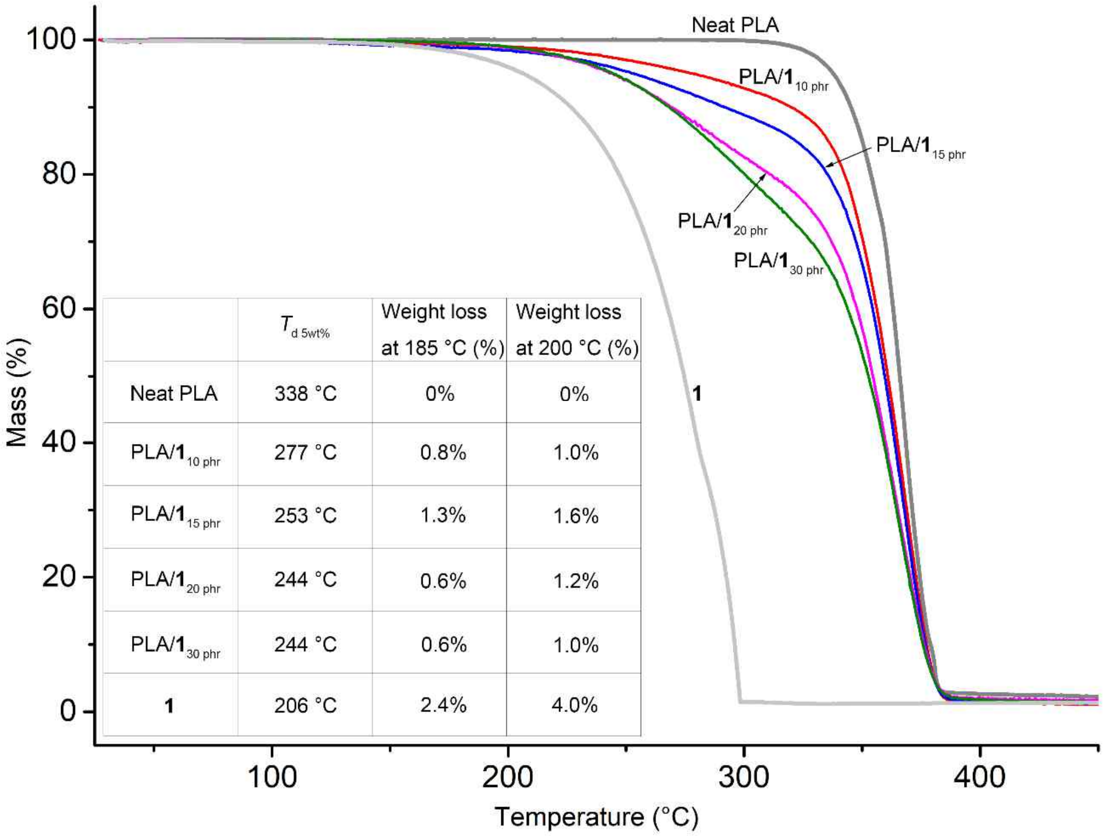

	$T_{d\ 5wt\%}$	Weight loss at 185 °C (%)	Weight loss at 200 °C (%)
Neat PLA	338 °C	0%	0%
PLA/**1**$_{10\ phr}$	277 °C	0.8%	1.0%
PLA/**1**$_{15\ phr}$	253 °C	1.3%	1.6%
PLA/**1**$_{20\ phr}$	244 °C	0.6%	1.2%
PLA/**1**$_{30\ phr}$	244 °C	0.6%	1.0%
**1**	206 °C	2.4%	4.0%

Figure S18. Full range DSC curves of PLA/**1** blends.

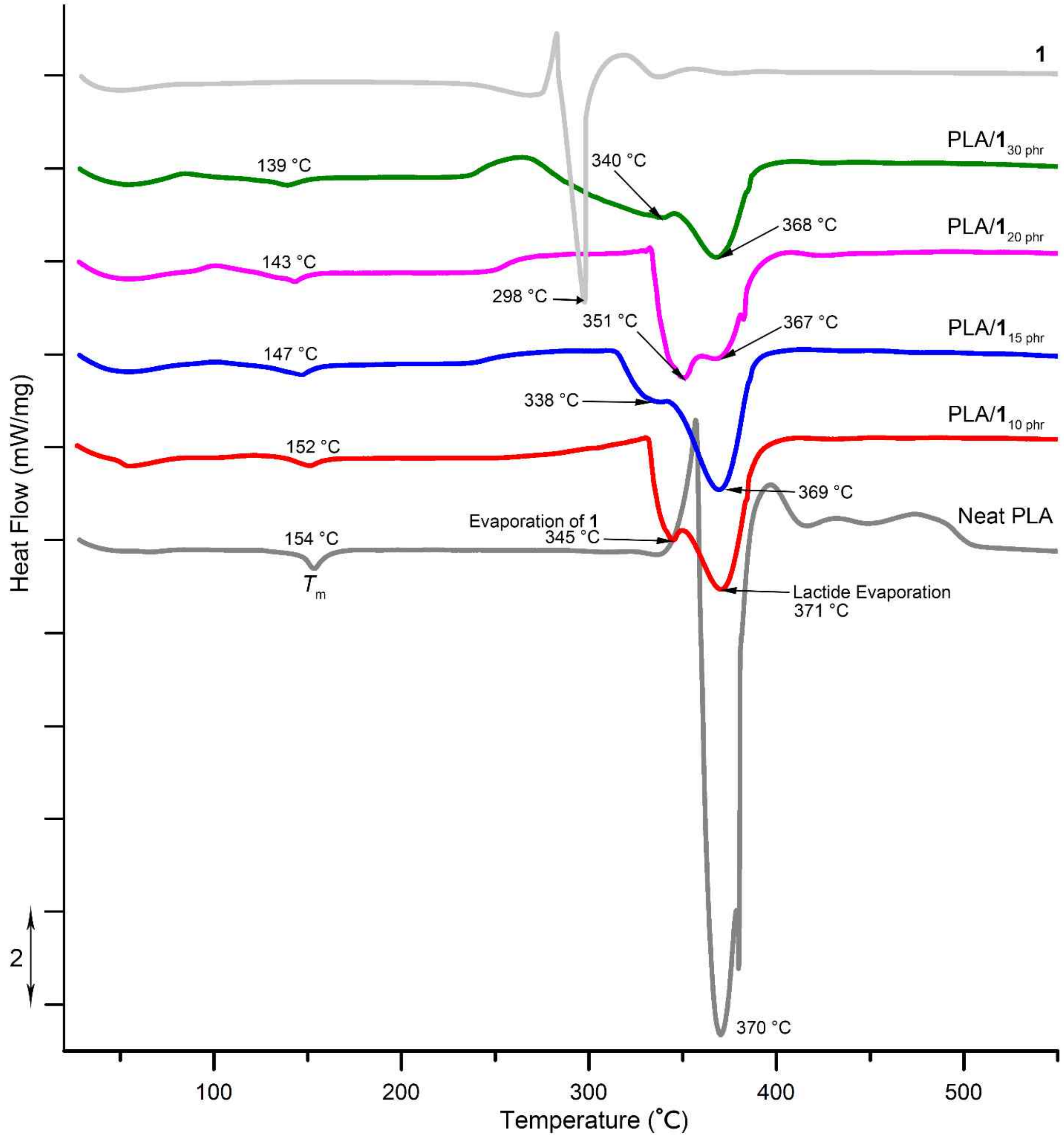

Figure S19. Dynamic moduli vs. angular frequency for PLA/**1**$_{10\ phr}$, PLA/**1**$_{20\ phr}$, and PLA/**1**$_{30\ phr}$ blends compared to neat PLA and PBAT measured using rotational rheometer at 170 °C.

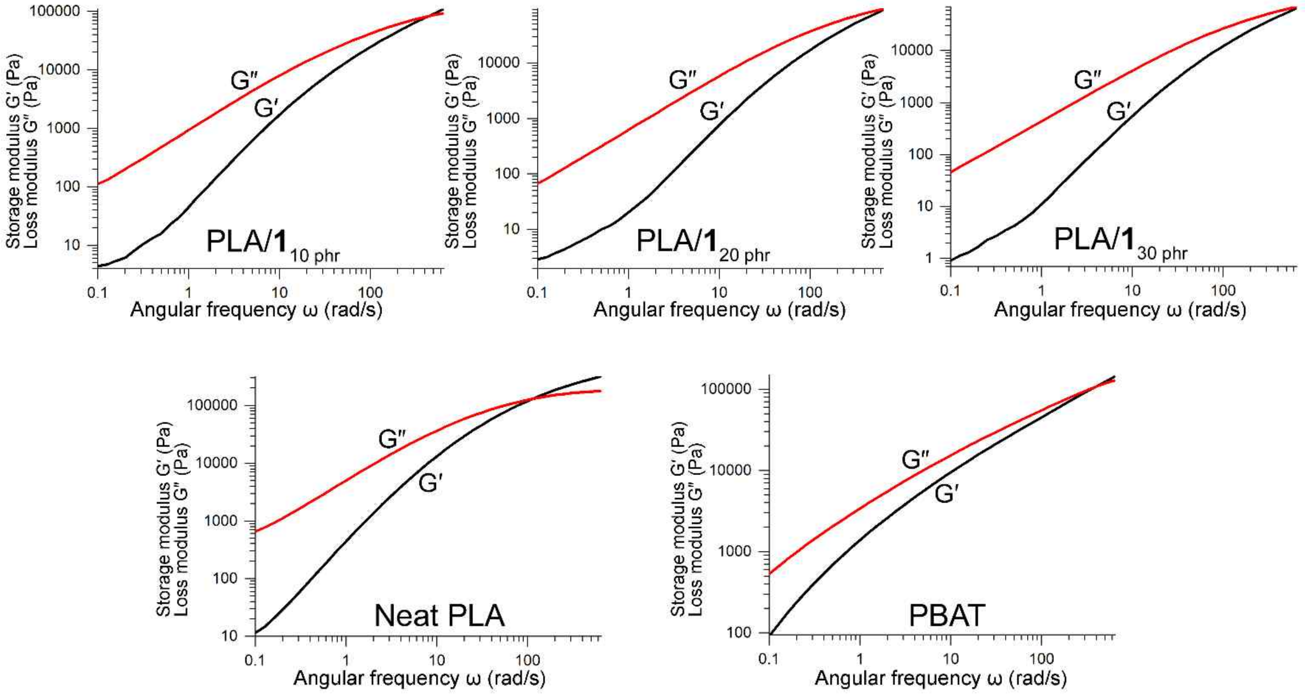

Figure S20. WAXD curves of unaged and aged specimens of (a) PLA/ATBC$_{20\ phr}$ and (b) PLA/**1**$_{20\ phr}$.

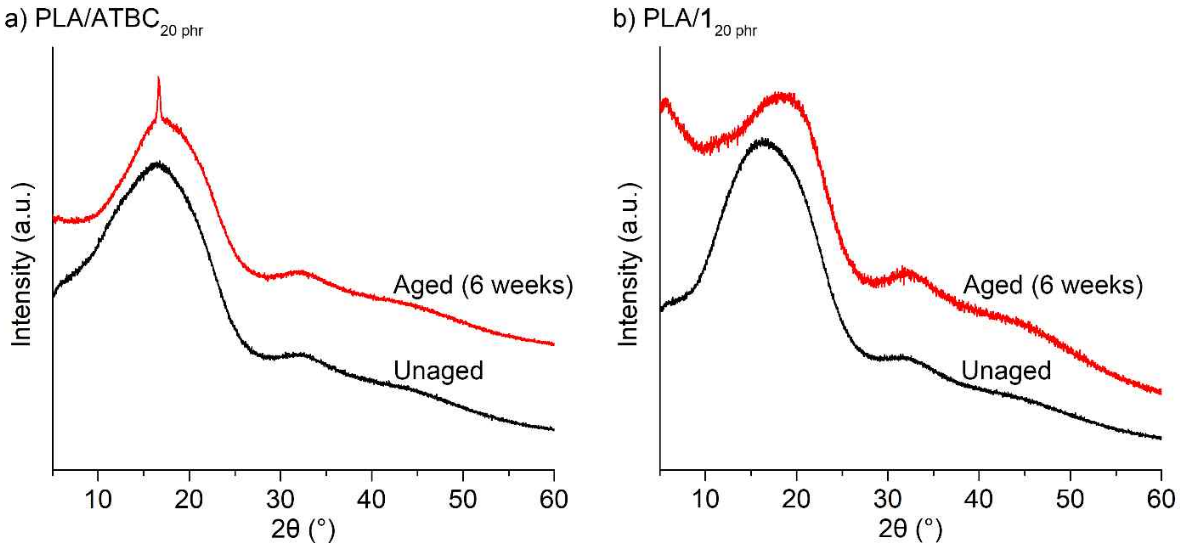

Figure S21. Biodegradability of (a) glycerol carbonate (**3**) and (b) propylene carbonate assessed by monitoring the amount of evolved CO_2 over time in a respirometer set to 25 °C and 50–55% water content with continuous air flow.

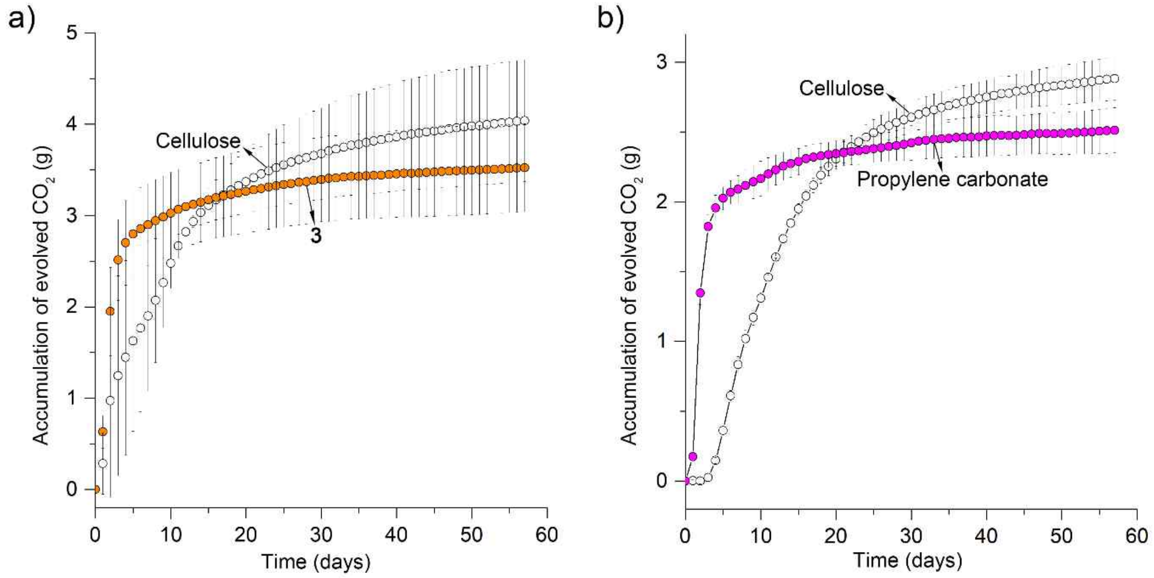

Figure S22. ^{1}H NMR spectra recorded to investigate stability of **2** in water (D_2O) over time.

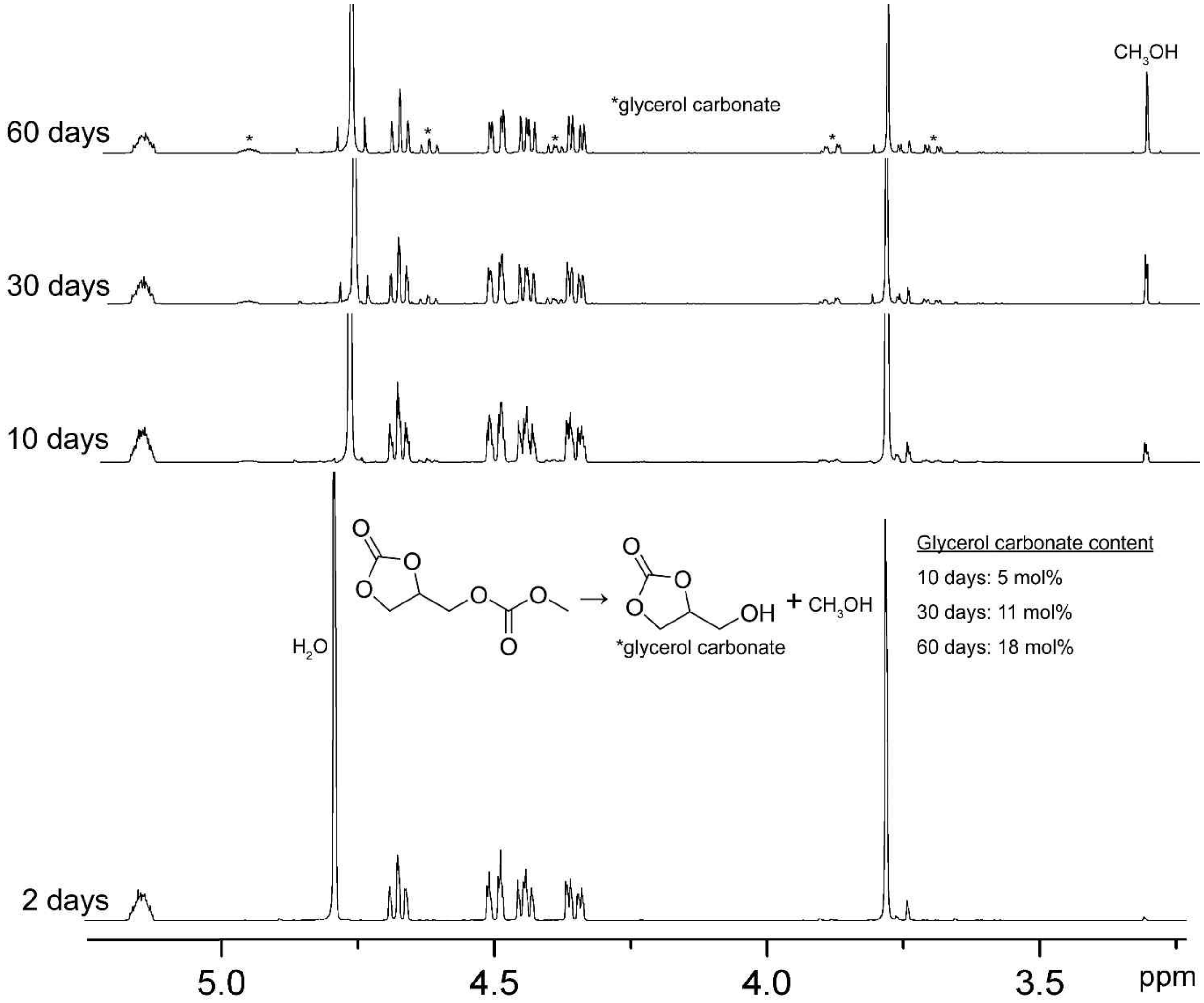

Figure S23. Custom-made reactor used for blending.

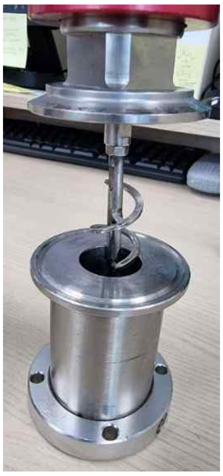

Supplement: RA-014-D3RA08922C-s001 [file RA-014-D3RA08922C-s001.pdf]
